# Genomic structure and evolution of the mating type locus in the green seaweed *Ulva partita*

**DOI:** 10.1038/s41598-017-11677-0

**Published:** 2017-09-15

**Authors:** Tomokazu Yamazaki, Kensuke Ichihara, Ryogo Suzuki, Kenshiro Oshima, Shinichi Miyamura, Kazuyoshi Kuwano, Atsushi Toyoda, Yutaka Suzuki, Sumio Sugano, Masahira Hattori, Shigeyuki Kawano

**Affiliations:** 1 Department of Integrated Biosciences, Graduate School of Frontier Sciences, University of Tokyo, Kashiwa, Chiba, Japan; 2 Department of Medical Genome Sciences, Graduate School of Frontier Sciences, University of Tokyo, Kashiwa, Chiba, Japan; 3 Faculty of Life and Environmental Sciences, University of Tsukuba, Tsukuba, Ibaraki, Japan; 4 Graduate School of Fisheries and Environmental Sciences, Nagasaki University, Nagasaki, Japan; 5 Center for Information Biology, National Institute of Genetics, Shizuoka, Japan; 6 Graduate School of Advanced Science and Engineering, Waseda University, Tokyo, Japan

## Abstract

The evolution of sex chromosomes and mating loci in organisms with UV systems of sex/mating type determination in haploid phases via genes on UV chromosomes is not well understood. We report the structure of the mating type (MT) locus and its evolutionary history in the green seaweed *Ulva partita*, which is a multicellular organism with an isomorphic haploid-diploid life cycle and mating type determination in the haploid phase. Comprehensive comparison of a total of 12.0 and 16.6 Gb of genomic next-generation sequencing data for mt^−^ and mt^+^ strains identified highly rearranged MT loci of 1.0 and 1.5 Mb in size and containing 46 and 67 genes, respectively, including 23 gametologs. Molecular evolutionary analyses suggested that the MT loci diverged over a prolonged period in the individual mating types after their establishment in an ancestor. A gene encoding an RWP-RK domain-containing protein was found in the mt^−^ MT locus but was not an ortholog of the chlorophycean mating type determination gene *MID*. Taken together, our results suggest that the genomic structure and its evolutionary history in the *U*. *partita* MT locus are similar to those on other UV chromosomes and that the MT locus genes are quite different from those of Chlorophyceae.

## Introduction

Sexual reproduction systems in eukaryotes can be divided into two types, in terms of determining sex/mating type in the haploid phase (UV systems) or in the diploid phase (XY/ZW systems)^1^. In the XY/ZW systems of mammals, insects, and plants, the structures of XY/ZW chromosomes and their evolution correspond reasonably well with predictions based on population genetics theory, whereby the suppressed recombination of the two chromosomes results in degeneration through Muller’s ratchet, background selection, the Hill-Robertson effect with weak selection, and the “hitchhiking” of deleterious alleles along with favorable mutations^2,3^. These theoretical predictions are constructed under the postulate that both sex chromosomes (XY or ZW) are heterozygous in the diploid phase and that they are distributed separately into the gametes (egg and sperm) via meiosis. In this case, deleterious mutations in an allelic gene on a sex chromosome, referred to as a gametolog, are masked by the counterpart gene on the other sex chromosome, resulting in sex chromosome degeneration driven by the above population genetic mechanisms of gene fixation. Sex chromosomes undergo stepwise degeneration, such as a size decrease, gene loss, accumulation of transposable elements, and decrease in codon bias, at different evolutionary times, resulting in “evolutionary strata”^1,4,5^. Several recent studies about plant Y chromosomes suggest that purifying selection influences their degeneration^6^. On the other hand, this postulate is not applicable to organisms with UV systems in which mutations in both sex chromosomes, named UV chromosomes, are not sheltered, because they have no allelic counterparts in the dominant haploid phase, leading to the expectation of different evolutionary patterns for UV chromosomes^7^. Recent simulations of UV systems suggest that the degeneration of sex chromosomes due to the accumulation of deleterious mutations by reduced recombination at mating type (MT) loci or sex-determining regions (SDRs) should be slower than in diploid determination systems because of the absence of masking of these mutations in the haploid phase; their differentiation would be driven by balancing selection, which involves maintenance of allelic genes in a population, to a greater extent than in XY/ZW systems^8^. However, there have been very few empirical studies of the structure and evolution of UV chromosomes.

**Figure 1:**
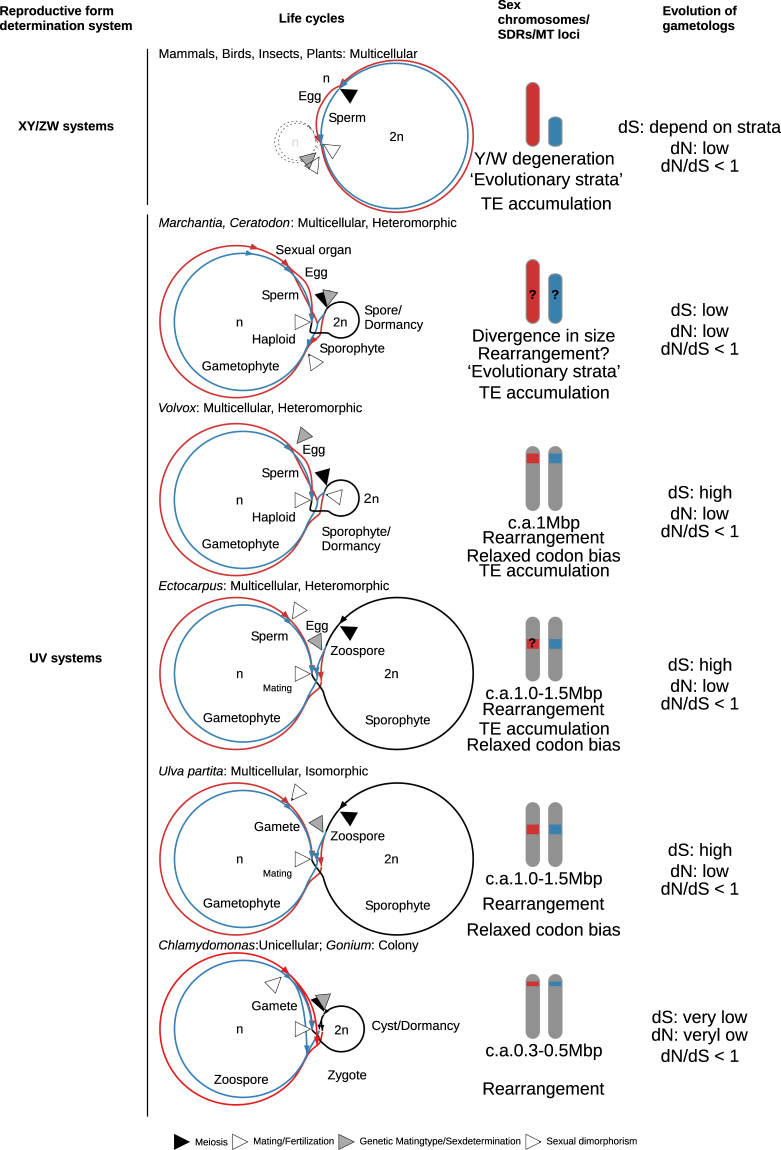
Life cycles and SDRs/mating type loci. Schematics of the life cycles of organisms are shown, along with the features of UV chromosomes. In XY/ZW systems, the diploid phase dominates, and the haploid phase is degenerate. In these organisms, sex determination occurs in the diploid phase, and a sex chromosome of a particular sex degenerates. In UV systems of multicellular organisms, the diploid saprophyte generates haploid zoospores or spores via meiosis, and mating type or sex is genetically determined by the UV chromosomes. Spores or zoospores with U and V chromosomes are female and male, respectively, and they develop haploid gametophytes. In UV systems of unicellular organisms, diploid zygospores after fusion of gametes with opposite mating types, some of which are dormant, undergo germination and meiosis to generate haploid vegetative cells that reproduce themselves and generate gametes under particular conditions, such as nutrient starvation. Genomic sequences of UV chromosomes are revealed in two types of life cycle: dominating haploid phase (e.g., the multicellular green alga *Volvox* and the moss *Marchantia*) and haploid and diploid phases of equal dominance (e.g., the brown alga *Ectocarpus*). Degeneration of sex chromosomes is thought to be observed only in the moss, with the other organisms harboring sex-determining regions (SDRs) in the UV chromosomes. The green seaweed *Ulva partita* has a life cycle with even domination of haploid and diploid phases, but it is not oogamous, in which gametes differentiate into eggs and sperms. The biflagellate gametes are anisogamous, apart from their size and ultrastructure. *U*. *partita* develops isomorphic gametophytes with a tubular thallus, and somatic cells differentiate into gametes^24^. Gametes of opposite mating types fuse, and a zygote develops into a sporophyte that is identical to a gametophyte in terms of morphology. The sporophyte somatic cells differentiate into zoospores that have four flagellae and are slightly larger than the gametes. The gametes have two mating types, mt^−^ and mt^+^, defined by the inheritance of chloroplast DNA from the mt^+^ gamete to the zygote^23,24^. Thus, the sexually reproductive form of this species is the mating type. The other type of MT locus is found in the unicellular green alga *Chlamydomonas* and the colonial green alga *Gonium*. Their MT locus sizes are smaller than the SDRs in the UV chromosomes, and they have a low diversity of gametologs, which are genes shared between the MT loci of individual mating types.

In the green plant lineage, the genomic sequences of MT loci and SDRs on UV chromosomes have been reported in four species: the unicellular green alga *Chlamydomonas* (Chlorophyta), the colonial green alga *Gonium* (Chlorophyta), the multicellular alga *Volvox* (Chlorophyta), and the liverwort *Marchantia* (Marchantiophyta)^9–11^. The three green algal MT loci and SDRs have been compared along with the evolution of multicellularity and oogamy because these algae evolved from an ancestral unicellular green alga, similar to *Chlamydomonas*, into *Volvox* with multicellularity and oogamy since least 200 million years ago (Mya)^12^. The sizes of *Volvox* male and female SDRs are over 1.0 and 1.5 Mb at the distal ends of the UV chromosomes and contain 70 and 80 genes, respectively, and they are larger than those of the of *Chlamydomonas* and *Gonium* MT loci, which are 200–300 kb and 360–500 kb and contain 40–41 genes and 24 genes, respectively^9,10^. Many genes of the *Volvox* SDR are the same as those located inside and outside of the *Chlamydomonas* MT locus, suggesting that expansion of the MT locus involves the surrounding genes^9^. These green algal MT loci/SDRs show high degrees of gene rearrangement^9,10,13^. Other well-studied green lineages include bryophyte species, specifically the liverwort *Marchantia* (Marchantiophyta) and the moss *Ceratodon* (Bryophyta). *Marchantia* has accumulated repeats in sex chromosomes, and gametologs are exposed to purifying selection^11,14,15^. Although the genomic sequences of *Ceratodon* have not been reported, population genetics and molecular evolutionary approaches indicate that non-recombination of SDRs exposes gametologs^16^. The genomic sequences of the MT loci have been reported in several species outside the green lineages. The brown alga *Ectocarpus* has a non-recombining SDR in which gametologs are exposed^17^. In fungi, the filamentous self-fertilized ascomycete *Neurospora tetrasperma* and the anther-smut fungus *Microbotryum lychnidis-dioicae* have UV chromosomes called “mating type chromosomes”; both the former and latter show an early stage of MT locus degeneration with two inversions via transposable elements over a 1.2–5.3 Mb region and a highly divergent MT locus with rearrangement over^18–20^. In both cases, degeneration signals, such as transposable element accumulation and relaxed codon bias, are found, but there are no clear evolutionary strata. These studies of the MT locus/SDR sequences provided both similar and contrasting findings (Fig. 1). The similar results included low levels of MT locus/SDR recombination, rearrangement of gametolog locations, exposure of most gametologs to purifying selection, low gene density, and gene loss. The contrasting results were a size range of 200 kb to 4 Mb, lack of clear strata except in *Ceratodon*, lack of relaxed codon usage bias in *Chlamydomonas* (but this has not been estimated in some species), and lack of accumulation of some transposable elements. The rules governing the generation of these differences are not yet clear.

Green seaweeds of the Ulvophyceae are multicellular and grow in coastal areas worldwide^21,22^. *Ulva partita* is a species of the Ulvophyceae and shows representative features of the life cycle of this order (Fig. 1). The species exhibits a typical haploid-diploid life cycle with alternating haploid and diploid phases, and the gametes have two mating types, mt^−^ and mt^+^^23,24^. Our previous study indicated a difference between the mating types, as evidenced by the arrangement of a putative mating structure involved in the fusion of gamete cells and an eyespot required for the recognition of photons; the putative mating structure and eyespot are arranged on opposite sides in mt^−^ gametes and on the same side in mt^+^ gametes^25^. The asymmetry between the mating structure and the eye spot is observed even in the isogamous green alga *Chlamydomonas reinhardtii* ^26^. Thus, *U*. *partita* develops a multicellular body and produces gametes with the determination of mating types in the haploid phase. *Ulva* species are anisogamous but not oogamous. *U*. *partita* develops isomorphic gametophytes and sporophytes with a thallus (leaf-like) shape, and their somatic cells differentiate into biflagellate gametes and tetraflagellate zoospores, respectively^23,24^. Compared with the other previously analyzed organisms with UV systems, *U*. *partita* may provide several insights into the drivers of MT locus/SDR evolution in terms of the life cycle. Isomorphism between gametophytes and sporophytes is expected to restrict the functions of the MT locus genes because they must function equally in the haploid gametophyte, haploid gamete, diploid sporophyte, and haploid zoospore. Natural populations of *Ulva* species show no dominance of haploid or diploid phases and no sexual bias between seasons, suggesting that isomorphism and sexuality do not affect fitness in either phase^27^. This is distinct from other organisms. For example, the two mosses develop extremely heteromorphic gametophytes and sporophytes or egg, sperm, and spores, and not all SDR genes are necessarily required for both phases, resulting in evolutionary relaxation of selective pressure on particular genes. *Ulva* genetically determines mating type after meiosis by harboring individual UV chromosomes in gametophytes, and it may acquire a transcriptional regulation system between mating types at the gamete stage or during gametogenesis.

Chlorophyta contains several classes; the major classes are Prasinophyceae, Trebouxiophyceae, Chlorophyceae, and Ulvophyceae^28^. In all Chlorophyta, the only known sex- or mating-determining gene is the *Chlamydomonas MID* (*Mi* *nus dominance*), encoding a putative transcription factor containing an RWP-RK domain, including a leucine zipper-like motif^29,30^. A *MID* ortholog has been found in the *Volvox* SDR, and its genetic manipulation results in the transformation of sex, from female to male or from male to female. However, the expression level of this gene is constant during spermatogenesis in males, suggesting that this gene does not play a role in sex determination but instead has a male-specific function in the differentiation of male vegetative cells into sperm^31^. *MID* is highly conserved in the Chlorophyceae lineage, but it is unclear whether other green algal lineages also possess this gene. With regard to green algal evolution, it is of interest to examine the conservation of MID among the distinct taxonomic classes Chlorophyceae and Ulvophyceae.

Here, we report identification of the MT locus in a species with a haploid mating type determination system without oogamy. The primary issue that this study aims to resolve is how much the genomic structures and evolutionary history of the MT locus in *U*. *partita* resemble those of the SDRs in UV chromosomes. The mating type determination system of *U*. *partita* is similar to the UV system in terms of the timing of mating determination, while the genes on the MT locus of the two mating types coexist in the diploid phase over a longer time scale. The isomorphism between the gametophyte in the haploid stage and the sporophyte in the diploid stage may affect the evolution of the MT locus. We also investigated the orthologs among the *U*. *partita* MT locus, the MT locus in *Chlamydomonas*, and SDRs in *Volvox*.

## Results

### Structure of the MT locus in the green seaweed *Ulva partita*

The PacBio long reads (1.7 × 10^6^ reads and 12.0 Gb for mt^−^ and 2.7 × 10^6^ reads and 16.6 Gb for mt^+^) from the genomes of both mating types were assembled into scaffolds. Although comparison of the scaffolds with unassembled PacBio long reads revealed mating type-specific (MTS) PacBio long reads, these reads were distributed over many scaffolds (Supplementary Tables 1 and 2; Supplementary Fig. 1). To select the MTS PacBio long reads located within a particular narrow region, the ratio of the sum of the lengths of 5–15 successive MTS PacBio long reads on the same scaffold per genomic length to that of the distal positions of the successive reads was determined (Supplementary Fig. 2). For reads located close together in a narrow region, the ratio reached 1 (see Supplementary Text for detailed analysis). This analysis identified a scaffold (# 632) containing a region that was highly divergent between the two mating types (Supplementary Fig. 3). In addition, the mapping results of the Illumina short reads from the two mating type genomes and RNA-sequencing (RNA-seq) reads derived from gametes and gametophytes were mapped, and the gene models predicted by the RNA-seq assemblies are shown in Supplementary Fig. 3. The highly adjacent MTS region was located in the middle of mt^−^ scaffold 632 over ~1.0 Mb (designated as the mt^−^ MT locus), and this region was particularly well mapped using short-read nucleotide sequences generated from the mt^−^, but not the mt^+^, genome and RNA-seq reads (Supplementary Fig. 3, 4^th^ and 5^th^ lanes). In addition, two highly adjacent MTS regions (mt^+^ MT locus) were identified for mt^+^ (scaffolds 4 and 898; Supplementary Fig. 2D–F, Supplementary Figs 4 and 5). These regions had lower gene density than the surrounding regions (8.2 and 5.8 genes/100 kb for the mt^−^ MT locus and the mt^+^ MT locus, and 10.6 and 9.8 genes/100 kb for the regions around these loci, respectively).

**Figure 2:**
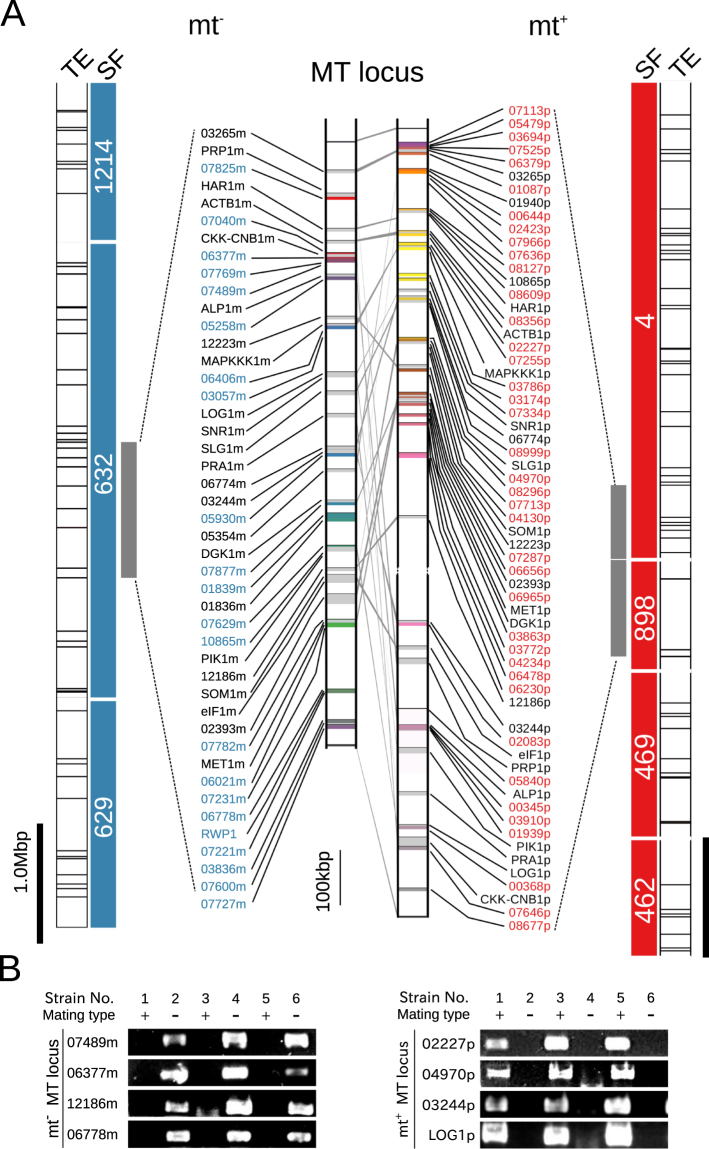
Genomic structures of the mating type (MT) locus in the green seaweed *Ulva partita*. (**A**) Scaffolds, transposable elements surrounding the MT locus, the MT locus in the green seaweed *U*. *partita*. SF, the MT locus scaffolds and neighboring scaffolds in both the mt^+^ and mt^−^ strains are shown. Blue and red bars indicate mt^−^ and mt^+^ scaffolds, respectively. Numbers are scaffold numbers. Transposable elements (TEs) of the same type are indicated by the same colors. The predicted MT locus genes were mapped on both genomes, and the positions of the 23 gametologs were compared between the mt^+^ and mt^−^ strains. The colored vertical bars indicate individual mating type-specific genes. Light gray vertical bars indicate mating gametologs. mt^−^, mating type minus; mt^+^, mating type plus. (**B**) Genomic PCR of MT locus genes for distinct wild-type strains. The presence of the *U*. *partita* MT locus genes in the MGEC-2 (mt^−^) and MGEC-1 (mt^+^) strains was confirmed in four other strains isolated from different areas along the Japanese coast. Genomic PCR was performed using primer sets for four genes in individual MT loci. PCR products of same primer sets were loaded on different gels (Supplementary Fig. 10). After electroporation, PCR products were visualized by ethidium bromide (EtBr) staining method. Captured EtBr fluorescence images of gels were cropped and images with low intensity were enhanced.

Using mt^−^ scaffold 632 and mt^+^ scaffolds 4 and 898, homologous scaffolds for the opposite mating type genome were identified based on a reciprocal homology search of scaffolds, which revealed that all had complementary scaffolds, and the two mt^+^ scaffolds were estimated to be a single fragmented mt^+^ MT locus (Supplementary Fig. 6). The mt^−^ and mt^+^ MT loci, together with the surrounding complementary regions identified in the flanking scaffolds, extended for 7.19 and 7.33 Mb, respectively (Fig. 2A).

As no genome of *Ulva* relatives has yet been analyzed, there are no training data for gene prediction based on genome sequencing data. Thus, for precise prediction of genes based on expression, sets of RNA-seq assemblies from gametes and gametophytes of the individual mating types were assembled and mapped on the scaffolds in and around the MT locus. The sets of RNA-seq assemblies were gathered based on homology, and then defined as genes. These analyses indicated that the mt^−^ MT locus and mt^+^ MT locus contained 46 and 67 mRNA-coding loci, respectively; several of the loci were assumed to generate splicing variants (Supplementary Tables 4 and 5).

Comparisons of the genes in the mt^−^ MT locus and the mt^+^ MT locus by reciprocal BLASTX analysis showed that 23 genes were shared by the two regions (Supplementary Table 6). These genes were defined as gametologs and were used to compare the genomic structures of the two regions; the results indicated that the mt^−^ and mt^+^ MT loci were highly rearranged and contained many MTS reads (Fig. 2A; Supplementary Figs 3–5).

In XY and ZW systems, particular transposable elements accumulate in the sex chromosomes^32^. No such accumulation of transposable elements has been detected in the MT loci of *Chlamydomonas* and *Gonium*, but it has been found in *Volvox* or *Ectocarpus*, *Marchantia* ^9,11,17^. Transposable elements were predicted based on homology with known transposable elements and comparison of the genome with itself. The results showed that transposable elements were present at the MT locus but were not more highly accumulated than in neighboring regions (mt^−^, MT locus: 0.32 ± 0.56/100 kb; neighboring region: 0.57 ± 0.84/100 kb; mt^+^, MT locus: 0.61 ± 0.85/100 kb; neighboring region: 0.62 ± 0.81/100 kb) (Supplementary Fig. 7).

*U*. *partita* has no genetic marker for estimating homologous recombination. Thus, a genomic PCR analysis was performed using four MT locus genes in both mating types for the two genome-sequenced strains and four other strains (mt^−^, MGEC-3 and 5; mt^+^, MGEC-4 and 6) isolated from different areas along the Japanese coast (Supplementary Table 6). All examined genes were linked to the mating types of the individual isolates (Fig. 2B), suggesting that these two regions contain the characteristics expected of an MT locus.

Finally, to examine the linkage between mating type and the identified MT locus, the mt^−^ strain, which was a different isolate than the one for which the genome was sequenced, was crossed with the mt^+^ strain, and the linkage between the mating types of their progeny and the unique gene markers of the MT locus was examined (Supplementary Table 7). A total of 10 of 16 progeny were mated with an mt^+^ tester strain, and 7 of 16 progeny were mated with an mt^−^ tester strain. Eight of the mt^−^ progeny harbored only mt^−^ MT locus markers, but two of them harbored both types of MT locus marker. In addition, six of the mt^+^ progeny harbored only mt^+^ MT locus markers.

### Evolution of gametologs in the *U*. *partita* MT locus

**Figure 3:**
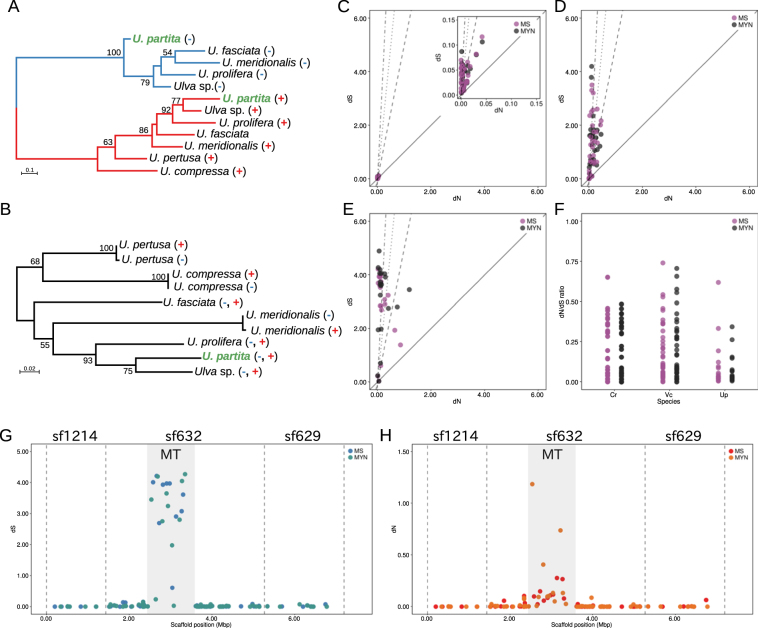
Evolution of the gametologs at the mating type (MT) locus. Molecular phylogenies of a gametolog in and a gene outside the MT locus. (**A**) Genes orthologous to an MT locus gametolog (encoding proliferation-associated protein 1, PAR1) from other species of the order Ulvales and (**B**) those of a gene outside the MT locus (encoding G-strand telomere-binding protein 1, GTBP1). The mating type of each strain of the species was determined previously (see Supplementary Table 7) and is indicated after the species name. Bootstrap values were obtained from analyses of 100 pseudoreplicates and are shown close to the branches (>50). The numbers above the scale bar indicate nucleotide substitutions per site. (CF) Synonymous (dS) and non-synonymous (dN) substitution rates of gametologs between the mt^+^ and mt^−^ strains of green algae. (**C**) dS and dN values for *C*. *reinhardtii*. (**D**) dS and dN values for *Volvox carteri*. (**E**) dS and dN values for *Ulva partita*. (**F**) dN/dS ratios of three algae. Up, *U*. *partita*. Cr, *C*. *reinhardtii*. Vc, *V*. *carteri*. MS, model selection method. MYN, modified YN method. Lines show specific thresholds of the dN/dS ratio. To make the dN/dS ratio of a gametolog clearly understandable, useful thresholds of this ratio (1, 0.2, 0.1, and 0.05) are indicated as follows: continuous line, 1, dashed line, 0.2, dotted line, 0.1, and dashed and dotted line, 0.05. (**G**) dS values and (**H**) dN values of the gametologs and genes around the MT locus. dN and dS values of the gametologs and the genes around the MT locus were calculated and plotted according to their positions in the mt^−^ genome. Numbers after “sf” indicate the scaffold number. The shadowed region shows the MT locus. Dashed lines show the border of the scaffold.

To analyze the evolutionary history of the gametologs in the MT locus, the homologous sequences of an MT locus gene encoding proliferation-associated protein 1, PAR1, and a gene encoding G-strand telomere-binding protein 1, GTBP1, in a region neighboring the MT locus, were isolated from species related to *U*. *partita*. Molecular phylogenies were then reconstructed (Fig. 3A and Supplementary Table 7). In all species examined, two types of homologous sequence were identified from the two distinct mating types, and the phylogenetic tree showed that the genes could be classified into two clades (Fig. 3A). In addition, these two clades were associated with the previously determined mating types (Supplementary Table 7). In contrast, the neighboring-region genes in each species were almost identical and were not classified into different clades in the molecular phylogeny (Fig. 3B). These data suggest that the investigated gametolog existed in the MT locus when this locus was established and evolved independently within the MT loci of the individual mating types.

Next, to determine the type of selective pressure exerted on the gametologs after their divergence, the nucleotide substitution rates at synonymous and non-synonymous sites (dS and dN, respectively) were estimated. They were also compared with those for genes at the known MT locus of *Chlamydomonas* and in the SDR of *Volvox* ^9,13^ (Supplementary Tables 8 and 9). Mean distances between individual genes on the plot were also calculated as an index to compare the divergence of MT locus genes (Supplementary Tables 10–12). Among 23 genes with several mRNA variants defined as gametologs by BLASTX analysis, two (06550 m/01365p and 12186 m/06628p) did not have coding sequences (CDSs) that could be aligned between gametologs; thus, the CDSs of the other 21 gametologs were aligned.

Both maximum-likelihood and approximate methods showed that the synonymous substitution rates for the gametologs in *U*. *partita* were considerably higher than the non-synonymous rates, and the non-synonymous rate/synonymous rate (dN/dS) ratios were <1 for all genes except one (means were 0.16 ± 0.36 for the approximate method and 0.16 ± 0.40 for the maximum-likelihood method), suggesting that these genes have been exposed to negative selective pressure and that their functions are highly restricted (Fig. 3E,F and Supplementary Table 13).

Mean dS values were much higher in *U*. *partita* and *Volvox* than in *Chlamydomonas*, and those of *U*. *partita* were higher than those of *Volvox* (Supplementary Fig. 8 and Supplementary Table 13). In addition, the dN means of *U*. *partita* and *Volvox* were higher than that of *Chlamydomonas*, but the difference between *U*. *partita* and *Volvox* was small.

Means of all distances between the two dots for each estimation method were calculated as an index of scattering (Supplementary Tables 10–13). If the mean distance is short, the dN/dS ratios would be expected to be homogeneous. The mean distances for *U*. *partita* in the two estimation methods were 1.43 ± 1.19 and 1.59 ± 1.23 (Supplementary Table 10). All mean distances were lower in *Chlamydomonas* than in *U*. *partita*, while some of the dN/dS ratios were slightly higher (0.17 ± 0.18 for the approximate method and 0.17 ± 0.17 for the maximum-likelihood method) and were plotted in a small area (mean distances were 0.03 ± 0.02 for the approximate method and 0.03 ± 0.02 for the maximum-likelihood method; Fig. 3C,F; Supplementary Table 11). Mean distances in *Volvox* were similar to those of *U*. *partita* (1.20 ± 0.92 for the approximate method and 1.10 ± 0.98 for the maximum-likelihood method), but comparison of the standard deviations showed that the divergence of their ratios was similar to that of *Chlamydomonas* (0.21 ± 0.22 for the approximate method and 0.22 ± 0.24 for the maximum-likelihood method; Fig. 3D,F; Supplementary Table 12). The molecular phylogeny and nucleotide substitution rates suggest that the *U*. *partita* gametologs were present at a common MT locus before the divergence of the relatives and that this region experienced a prolonged period after separation.

The synonymous and nonsynonymous substitution rates of the genes around the MT locus were estimated by the two methods. From the scaffold data around the MT loci of mt^−^ and mt^+^, the CDSs of the genes were extracted and their associations were determined using BLASTN. After estimation of the synonymous and nonsynonymous substitution rates of 119 genes by the two methods, both values were plotted according to the mt^−^ positions of the MT locus (Fig. 3G,H). The data showed that almost all synonymous and non-synonymous substitution rates of the genes around the MT locus were near zero or zero; additionally, the synonymous substitution rates were higher than those of the MT locus, and the non-synonymous substitution rates were slightly higher than those of the MT locus.

It has been reported that relaxed codon usage bias occurs with reduced recombination in sex chromosomes^33,34^. Furthermore, the codon usage in CDSs obtained from all mRNA data and the codon usage for the MT locus genes were compared with those of other autosomal locus genes (Supplementary Table 14). The codon usage patterns of all of the autosomal genes and the MT locus genes did not appear to differ (Supplementary Fig. 9), and comparisons between them and those of mt^−^ and mt^+^ autosomal locus genes showed high correlations (Poisson’s correlation, 0.98; p-value, 2.2 × 10^−16^). However, the codon usage of half of the MT locus genes in both mating types differed significantly from that of the autosomal genes, and these included the gametologs, which showed a low dN/dS ratio (Supplementary Table 14).

### Motifs and molecular phylogeny of RWP-RK domain-containing proteins in the MT locus and autosomes

We investigated whether there were orthologs among the *U*. *partita* MT locus genes, the *Chlamydomonas* MT locus, and the *Volvox* SDR. Although no gene was clearly shared among the MT loci and SDR in the three species, very weak homology with *MID* was found in a gene only in the *U*. *partita* mt^−^ MT locus, named *UpaRWP1*. To assess the relationship between *MID* and *UpaRWP1*, a BLAST analysis was performed using all *Chlamydomonas* RWP genes as queries against the entire *U*. *partita* mRNA database from the assembly of RNA-seq data. Two of the autosomal RWPs (*UpaRWP2* and *UpaRWP3*) were identified and mapped to locations other than the MT locus. In addition, genes encoding proteins containing the RWP-RK domain in Chlorophyta were collected from the annotated genes from the genomes of five species: *Chlamydomonas reinhardtii*, *Volvox carteri*, *Gonium pectorale*, *Coccomyxa subellipsoidea*, and *Micromonas pusilla*. Although these genes encode proteins containing a single RWP-RK domain, the protein lengths are very diverse.

**Figure 4:**
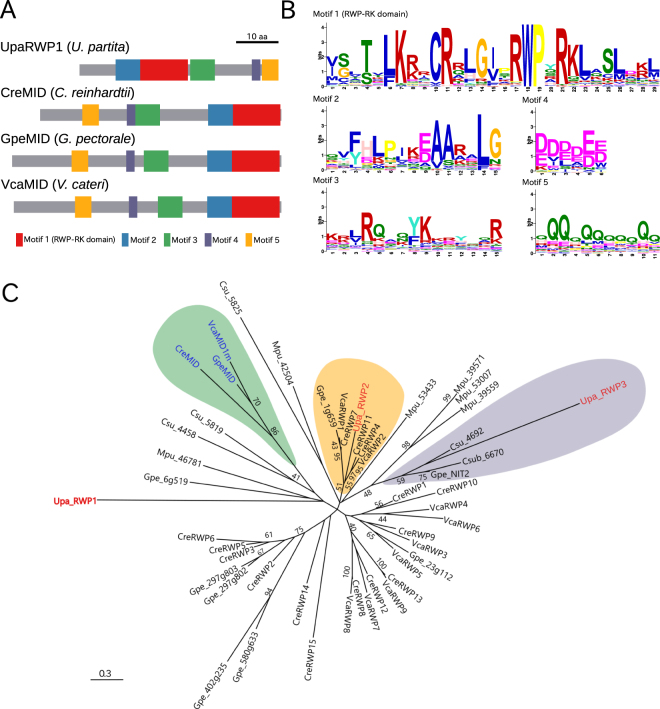
Conserved motifs and molecular phylogeny of *U*. *partita* RWP1, Volvocales MIDs, and RWP-RK domain-containing proteins in green algae. (**A**) Schematic of the protein structure of *U*. *partita* RWP1. Among proteins containing the RWP-RK domain in Chlorophyta, conserved motifs were identified by using MEME, and the motifs of *U*. *partita* RWP1 and Volvocales MIDs are shown by distinctly colored boxes, reflecting their positions and lengths. Gray bars show the total lengths of the individual proteins. In particular, Motif 1 contains RWPxRK sequences. Bar shows 10 amino acids. (**B**) Sequence logos of individual motifs. Information contents of individual amino acids at a position in each motif are visualized by the height of the capital letters designating amino acid residues. The *y*-axis represents the bit value, as information content, of which the maximum is log_2_20 ≈ 4.32. The *x*-axis represents the position of an amino acid residue in each motif. (**C**) Molecular phylogeny of the RWP-RK domain-containing proteins in Chlorophyta. From the deduced amino acid sequences of the proteins containing the RWP-RK domain in the genomes of *Ulva partita*, *Volvox carteri*, *C*. *reinhardtii*, *Gonium pectorale*, *Coccomyxa subellipsoidea*, and *Micromonas pusilla*, the amino acid sequences of motifs conserved in all proteins were combined in individual proteins, and the aligned combined sequences were used for the construction of an unrooted molecular phylogeny using the maximum-likelihood method. *U*. *partita* RWP1 (UpaRWP1) in the MT locus is shown in red. The highlighted genes indicated in green, orange, and purple are classified into clades containing Volvocales MIDs (blue), one of the *U*. *partita* autosomal RWP genes (UpaRWP2; accession ID, DN37992), and the other of the *U*. *partita* autosomal RWP genes (UpaRWP3; accession ID, DN130398), respectively. The prefixes before the gene symbols are abbreviations of the species names: Upa, *U*. *partita*, Cre, *C*. *reinhardtii*, Vca, *V*. *carteri*, Gpe, *Gonium pectorale*, Csu, *Coccomyxa subellipsoidea*, Mpu, *Micromonas pusilla*. Bootstrap values were obtained from analyses of 100 pseudoreplicates and are shown close to the branches (>50). The numbers below the scale bar indicate amino acid substitutions.

Conserved motifs among all of the deduced amino sequences were identified with the MEME program; five conserved motifs were identified (Fig. 4A,B). Motif 1 contained the RWPxRK sequence, which was conserved among all identified gene products. Three Volvocales *MID*s were very similar in terms of protein length and the order of the five motifs (Fig. 4A,B). Although the protein length of UpaRWP1 was similar to that of MID, the order of the five motifs differed. The order of Motif 1 and Motif 2 was conserved among UpRWP1 and the Volvocales *MID*s, but the order of the other motifs was not.

A molecular phylogenetic tree was constructed using all five motifs of UpaRWP1, two *U*. *partita* autosomal RWPs, MIDs, and other Chlorophyta RWPs (Fig. 4C). The three MIDs were classified into a clade with high statistical support, whereas UpaRWP1 was classified into a different clade from that containing the MIDs, albeit with low statistical support (Fig. 4C). In addition, an autosomal *U*. *partita* RWP (UpaRWP2) was classified into a clade containing *Chlamydomonas* RWP11 (CreRWP11) with high statistical support and few amino acid substitutions^35^. The other (UparRWP2) was classified into a clade containing NIT2, which is a regulator of nitrate assimilation^36^. Thus, UpaRWP1 differed not only from MIDs but also from the autosomal RWPs, suggesting that this gene is not an ortholog among the three species and was acquired independently in the *U*. *partita* MT locus and the two other species.

### Expression of MT locus genes in gametogenesis

**Figure 5:**
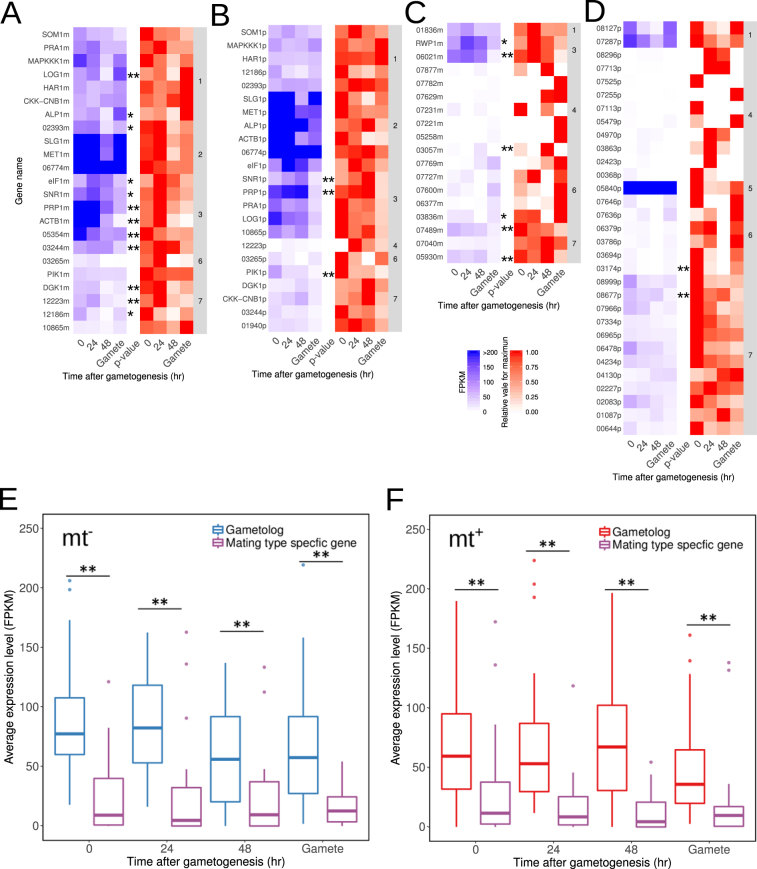
Expression changes in the mating type (MT) locus during gametogenesis. (**A**) Gametologs of mt^−^. (**B**) Gametologs of mt^+^. (**C**) Mating type-specific genes of mt^−^. (**D**) Mating type-specific genes of mt^+^. The blue heat map shows the average fragments per kilobase of exon per million fragments (FPKM) values from three biological replicate for gametologs and mating type-specific genes in each mating type during gametogenesis. High FPKM values (>200) are shown in the same color as values of 200. The red heat map shows relative values for maxima of FPKM values. The numbers in the right column are the numbers of clusters calculated by the k-means method. Results of one-way ANOVA for individual genes are shown on the right side of the heat maps (**p < 0.01, *p < 0.05). (**E**),(**F**) Average FPKM and values normalized to the beta-tubulin gene. Box-whisker plots of FPKM values for gametologs and mating type-specific genes. Results of Wilcoxon’s rank-sum test of all expression data between gametologs and unique genes during gametogenesis (**p < 0.01, *p < 0.05).

Mating type-specific genes are expected to provide genetic differences to opposite mating types after meiosis, and the expression of the MT locus genes may provide differentiation of gametophytes and gametes between mating types. On the other hand, there are no differences in gametophytes between mating types in *U*. *partita*, but there are some differences in gametes with asymmetry of mating structure and eye spot and mt^−^-specific fusion machinery^25,37^. To analyze the expression levels of the *U*. *partita* MT locus genes, RNA-seq data during gametogenesis from the two mating types with biological replications were mapped on the genome, and the expression levels of the MT locus genes were estimated. The expression levels of all splicing variants are shown with the results of one-way ANOVA for four time points and the Dunnett test for multiple comparisons between gametophytes before induction and at various time points after gametogenesis (Supplementary Table 15). After removing splicing variants with low expression and clustering these data, the expression data of gametologs and mating type-specific genes as well as the relative expression changes were plotted separately (Fig. 5A–D). The expression data showed that most of the genes, including both the unique genes and the gametologs, were expressed constantly during gametogenesis in both mating types. In addition, the expression levels of gametologs were much higher than those of the mating type-specific genes (Fig. 5E,F).

Statistical analyses showed that the expression levels of 18 and 6 genes changed significantly during gametogenesis in mt^−^ and mt^+^, respectively. Among the 18 mt^−^ MT locus genes, the two gametologs *LOG1m* and *ALP1m* were upregulated in gametes; two gametologs (*DGK1* and *12223m*) and two mating type-specific genes (*07489m* and *05930m*) were classified into cluster No. 7 and, except for *12223m*, were downregulated during gametogenesis; six gametologs (*elF1m*, *SNR1m*, *PRP1m*, *ACTB1m*, *05354m*, and *23244m*) and two mating type-specific genes (*RWP1* and *06021m*) were co-expressed (cluster No. 3), and, except for *05354m*, their expression levels increased at 24 h after gametogenesis and decreased to the pre-gametogenesis level in gametes (Fig. 5A,C). Of the mating type-specific genes at the mt^−^ MT locus, the expression level of *03057m* was increased at 48 h after gametogenesis and then decreased to zero in gametes; the others were downregulated in gametes. Among the 6 mt^+^ MT locus genes, two gametologs (*SNR1p* and *PRP1p*) were classified into co-expression cluster (#3, see above); one other gametolog (*Pik1p*) and two mating type-specific genes (*03154p* and *08677p*) were downregulated (Fig. 5B,D). In summary, among the genes significantly upregulated in mt^−^, thirteen mt^−^ genes and two mt^+^ genes were significantly upregulated during gametogenesis or in gametes, and the others were downregulated.

## Discussion

### Comparison of the genomic structures of the *Ulva partita* MT locus with those of other organisms

In this study, we used a third-generation sequencing technology with a single-molecule sequencing method to identify the putative mating locus in the genome of the green macroalga *Ulva partita*. The size of the *U*. *partita* MT locus (~1.0–1.5 Mb; Fig. 1 and 2) resembles that of the SDR of *Volvox*, which is a UV system with a dominating haploid phase in a life cycle showing phasic heteromorphism (sporophytes do not develop, and meiosis occurs in the zygote) and gamete dimorphism (eggs and sperm), and the brown alga *Ectocarpus*, which is also a UV system with a haploid-diploid life cycle with phasic heteromorphism and gamete dimorphism (motile and immotile gametes in males and females, respectively)^9,17^. These sizes are smaller than those in two fungal MT loci (*Neurospora* and *Microbotryum*) and larger than those in unicellular and colonial green algae (*Chlamydomonas* and *Gonium*)^9,10,18–20^. In addition, the gametolog location rearrangements in the individual mating types resemble not only the SDRs of *Volvox* and *Ectocarpus* but also the MT loci of all others sequenced to date. Therefore, genomic rearrangement in the MT loci and SDRs is a common phenomenon in haploid organisms. Accumulation of transposable elements and low gene content are found in the MT loci and SDRs of *Volvox*, *Ectocarpus*, and *Microbotryum* but not of *Chlamydomonas*, *Gonium*, or *Neurospora*. Note that the *Neurospora* locus is thought to be young and therefore to show less accumulation of transposable elements^18^. The *Ulva* MT locus showed lower gene content but not a high level of transposable element accumulation. The low gene content suggests chromosomal degeneration with gene loss, while the low level of transposable element accumulation may reflect the shortage of transposable element data for Ulvophyceae.

*Chlamydomonas* and *Gonium* are unicellular and colonial, respectively, with few cells in their gametophytes, and meiosis occurs in diploid spores^38^. This is the clearest difference from other organisms except the two fungi *Neurospora* and *Microbotryum*. On the other hand, the fungi *Neurospora* and *Microbotryum* exhibit automictic reproduction, which is a mating system involving a meiotic tetrad^20,39^. Such automictic reproduction is predicted to favor successive linkage to a set of mating type genes that experience deleterious and recessive mutations^40,41^. Therefore, the driver of evolution in fungal MT type loci may be distinct from those in the other organisms. While we initially expected to observe some differences between the *Ulva* MT locus and others, resulting from the contribution of types of diploid phases in UV systems, the sizes and structures seem to be associated with the multicellularity of gametophytes, except in the fungi, rather than equal dominance of the diploid phase. In the case of the liverwort, *Marchantia*, the UV chromosomes of which are thought to be dimorphic, complete genomic sequences have not yet been reported^11^. When available, they will likely provide some insight into the evolution of the MT locus/SDR in organisms in which the sex/mating type is determined in the haploid stages.

Although the MT locus genes were tightly linked to the mating types of the *U*. *partita* isolates, inheritance patterns from a sporophyte to gametophytes were somewhat unusual. Several progenies had the both mating type-specific genes, suggesting that they are diploid. However, these progenies mated with the mt^+^ tester strain. We now hypothesized that this is an apomixis-like phenomenon found in several organisms and will report on this in more detail in the future. In addition, the finding that the gametes of the diploid progenies mated with the mating type plus gametes is similar to an observation in *Chlamydomonas*, in which diploid gametes artificially generated by using auxotrophic mutants exhibited the mt^−^ phenotype^42^. This suggests that the MT locus gene(s) of *U*. *partita* may determine the mating type.

### Evolution of the *U*. *partita* MT locus

A comparison of the dS and dN values of the gametologs in the *U*. *partita* MT locus showed that the dS values were significantly higher than the dN values (Fig. 3). Although they are dependent on the substitution model and generation time, dS values are underestimated when they are greater than ~2^43^. In total, 15 dS values for the approximate method and 13 for the maximum-likelihood method were >2, indicating that nucleotide substitution at a given site may have occurred several times and that substitutions at some sites are saturated. Thus, the possibility that the dS values estimated from this data set are not accurate cannot be ruled out. In contrast, with the exception of one gene, all dN values were <1. This suggests that the dN/dS ratios for all gametologs, except one, are <1, although dS may be over- or underestimated, and that the genes have been exposed to purifying selection with a functional constraint.

*Volvox* is considered to have diverged from a unicellular ancestor, similar to *Chlamydomonas*, at least 200 Mya^12^. Comparison of the MT locus/SDR and neighboring autosomal genes between *Chlamydomonas* and *Volvox* suggested that the expansion from an ancestral MT locus to an SDR involving neighboring autosomal genes occurred with cooption of gene functions^12^. Although the timing of the establishment of the *U*. *partita* MT locus is currently unclear, the molecular phylogeny of a gametolog among *U*. *partita* relatives was associated with mating type, and the *U*. *partita* gametologs showed high proportions of synonymous substitutions (Fig. 3). There are no fossil samples available to calibrate the molecular clock of the order Ulvales, including *U*. *partita*, and it is therefore difficult to determine the timing of the establishment of the *Ulva* MT locus. However, the diversity of gametologs and the molecular phylogeny within Ulvales suggest that this locus was established at least at the origin of the examined species and has experienced a long period of evolution. The low diversity of the dN/dS ratio in the *U*. *partita* gametologs suggests that no such expansion during evolution of the *Volvox* SDR has occurred for a prolonged period, because newer gametologs would be expected to have lower dN and dS values than those of older genes if there had been expansion involving the addition of autosomal genes adjacent to the MT locus^9^; alternatively, rapid gene losses may have been occurring, and this may be related to a larger number of mating type-specific genes than are present in other green linage organisms. This is similar to the SDR of the UV chromosome in *Ectocarpus*, which was estimated to have been established more than 70 Mya^17^.

### Evolutionary relationship between *Chlamydomonas MID* and an MT locus gene encoding an RWP-RK domain

In the Chlorophyta, a gene determining mating type has been identified only in *Chlamydomonas*, namely, *Minus Dominance* (*MID*), which is located at the mt^−^ MT locus^29^. This gene encodes a putative transcription factor containing an RWP-RK domain, which includes a leucine zipper-like motif^29,30^. Although *MID* homologs have been found across the Volvocales and the *Volvox MID* (*VcaMID*) is located on the SDR of the V chromosome in males, genetic manipulation data and the constitutive expression of *VcaMID* in males during both vegetative and sexual stages suggest that this gene does not play a role in sex determination but instead has a sex-specific function in the differentiation of male vegetative cells into sperm^9,31,44^. One RWP-RK domain-containing gene was found only at the mt^−^ MT locus of *U*. *partita* and was named *RWP1* (Fig. 4). Genes containing the RWP-RK domain are present in the genomes of various plants, including green algae, and their orthologs in *Arabidopsis* play roles in the development of eggs and embryos^45–49^. Although *RWP1* is a potential determinant of mating type, transcriptome analysis showed that it is expressed even in gametophytes, with a slight increase at an early time point, and decreases to the initial level in gametes, suggesting that this gene is related to mating type differentiation at the transcriptional level (Fig. 5C). This is similar to the case of *Volvox MID* ^9^. If *RWP1* is a master gene for mating type determination, future studies should address whether post-transcriptional or post-translational regulation occurs during gametogenesis and, if so, which mechanisms underlie this process.

### Degeneration of *U*. *partita* MT locus genes

Expression levels of most of the MT locus genes were constant during gametogenesis in both mating types, and those of gametologs were much higher than those of mating type-specific genes (Fig. 5E,F). *Ectocarpus* sp. show much lower transcript abundance in haplotype mating type-specific SDR genes, and this may reflect degradation of the promoter and *cis*-regulatory sequences of these SDR genes^17^. This corresponds to the mating type-specific genes of *U*. *partita*. Although comparisons among species closely related to *U*. *partita* are required, these low levels of mating type-specific gene expressions may indicate their degeneration via mutations not only in protein-coding sequences but also in promoter regions. Degeneration is supported by the presence of degeneration signals such as relaxed codon usage bias in both gametologs and mating type-specific genes (Supplementary Table 14).

### Expression of *U*. *partita* MT locus genes

The low dN/dS ratios of gametologs suggest that their gene functions are conserved between mating types. The mating type-specific genes confer genetic differences between mating types after meiosis and may lead to dimorphism between them. *U*. *partita* shows no difference between opposite mating type gametophytes, and only structural differences were found between gametes. Constitutive expression and expression changes during gametogenesis in most of the MT locus genes indicate that their functions are identical during each stage. A group of mt^−^ gametologs (cluster No. 3) exhibited significantly increased expression levels at the early stage of gametogenesis. These genes encoded orthologs of actin (*ACTB1m*), alfin-like protein (*ALP1m*), small nuclear ribonucleoprotein polypeptide G (*SNR1m*), proliferation-associated protein 1 (*PRP1m*), and eukaryotic initiation factor (*eIF1m*) that are expected to be involved in important cellular functions, and their allelic genes, except *eIF1m* and *PRP1m*, did not show changes in expression levels, suggesting that these genes may modulate differentiation of gametes via transcriptional regulation. On the other hand, other mating type-specific genes (*RWP1* and *06021m*) were upregulated, and still others were downregulated; most of them, except *RWP1*, were shown to encode proteins with no homology to known proteins, making it difficult to predict their function(s) during gametogenesis. Our group and another team have developed a system to introduce and transiently express a transgene using a polyethylene glycol method. This method, as well as the application of other methods, including RNA interference and genome-editing technologies, will provide further information regarding the molecular functions of the MT locus and its constituent genes^50,51^.

In conclusion, we identified a locus linked to mating type in the green macroalga *U*. *partita* with an isomorphic haploid-diploid life cycle and without oogamy. This locus was highly rearranged and exhibited suppressed recombination for a prolonged period. In addition, the *U*. *partita* MT locus has features similar to UV chromosomes. Although the *U*. *partita* mt^−^ MT locus harbored a gene encoding a protein containing an RWP-RK domain, *RWP1*, which is found in the *Chlamydomonas* mating type determination gene *MID*, this gene is not an ortholog of *MID*. During gametogenesis, the expression level of *RWP1* increased once and then decreased in gametes as much as in gametophytes.

## Materials and Methods

### Algal materials and culture conditions

#### Algal strains

The pairs of mt^−^ (MGEC-2) and mt^+^ (MGEC-1) strains of *Ulva partita* used were collected from the coast of Japan (Supplementary Table 7)^23–25,52^. We recently renamed this species from *Ulva compressa* to *U*. *partita* based on its molecular phylogeny and morphology^35^. The *Ulva* strains were obtained from culture collections at Kochi University, and their mating types were determined previously based on gamete sizes^53–55^. The strains are maintained in the culture collection of Nagasaki University (Nagasaki, Japan).

#### Culture conditions

Laboratory cultivation and induction of gametogenesis were performed as described previously^23,56^. Briefly, thalloid gametophytes were grown at 16 °C under 150 µmol photons m^−2^ s^−1^ light under a 10 h:14 h light (L):dark (D) cycle in artificial seawater for 28 days. Then, 2 days after the induction of gametogenesis by rinsing several times and transferring to long-day conditions at 23 °C under 150 µmol photons m^−2^ s^−1^ light under a 14 h:10 h L:D cycle in seawater, migrating gametes were released from the gametophytes. Positive phototaxis was used to collect the gametes. This alga was cultured with symbiotic bacteria because inappropriate development occurs in the absence of symbiotic bacteria.

### Genome and RNA-sequencing

#### DNA isolation

To remove bacterial DNA contamination prior to genome sequencing, gametic cells were gathered by illumination using a natural white fluorescent light that induced positive phototaxis. Gametes of both mating types with a fresh weight (FW) of approximately 1.5 g were collected. The collected gametic cells were frozen in liquid nitrogen, ground, and subjected to genomic DNA isolation using a Plant Maxi Kit (QIAGEN, Venlo, The Netherlands).

#### RNA extraction

Gametic cells were collected by the same method as used to gather cells for RNA isolation. Gametophytic thalli were collected at 0, 24, and 48 h after the induction of gametogenesis, and three replicates of each mating type were included. Total RNA was extracted from 50 mg of gametic cells and gametophyte thalli using an RNeasy Plant Mini Kit (QIAGEN) according to the manufacturer’s protocol. Contaminating DNA was removed using RNase-Free DNase I (QIAGEN).

#### DNA and RNA-sequencing

Genomic sequences of *U*. *partita* MGEC-1 and MGEC-2 were determined using PacBio single-molecule real-time sequencing (long reads) and Illumina MiSeq for paired-end (PE) short reads. Briefly, for PacBio sequencing, a library was constructed using the PacBio DNA Template Prep Kit 2.0 (Pacific Biosciences, CA, USA) according to the manufacturer’s protocol. For Illumina sequencing, a library was constructed using the TruSeq- DNA LT Sample Prep Kit (Illumina, CA, USA). The PacBio sequencing and selection of reads of more than 500 bp provided 1.7 M reads of 12.0 Gb and 2.7 M reads of 16.6 Gb from the mt^−^ and mt^+^ genomes, respectively. The average lengths of mt^−^ and mt^+^ PacBio reads were 7182 and 6136 bp, respectively. The Illumina sequencing generated 271 M reads of 100 bp (totaling 27.1 Gb) and 252 M reads of 100 bp (totaling 25.2 Gb) from the mt^−^ and mt^+^ genomes, respectively. Sequences in the obtained long reads were corrected by mapping the short reads and comparing individual sites, and the corrected long reads were assembled into scaffolds using HGAP3 software (Pacific Biosciences, CA, USA). Finally, BLASTN analysis was performed using the scaffolds as query sequences against the RefSeq microbial genome database (http://www.ncbi.nlm.nih.gov/refseq/) with a threshold e-value of 1 × 10^−30^ to exclude contaminating sequences from the symbiotic bacteria. For mt^−^ and mt^+^ genomes, the final numbers of scaffolds after removing bacterial genome contamination were 851 and 1385, and the total lengths of scaffolds were 110.2 and 116.7 Mb. These sequences were used for later analyses. After assembly, the Illumina short reads were mapped onto the assembled sequences of both mating types. The proportions of properly paired reads were 99.8% and 99.5%, respectively. In addition, the proportions for mt^−^ scaffold 632 and mt^+^ scaffolds 4 and 898 were 99.2%, 99.1%, and 99.4%, respectively.

For RNA-seq, the purification of mRNA from total RNA and construction of a cDNA library from the purified mRNA were performed using TruSeq RNA Sample Preparation (ver. 2; Illumina). The cDNA library was sequenced using an Illumina HiSeq 2500 instrument. A summary of the reads obtained is shown in Supplementary Table 1.

#### Identification of mating type-specific long genomic sequence reads

The long reads and the scaffolds were used to identify mating type-specific genomic regions. First, the corrected long reads from mt^−^ were mapped to the scaffolds from mt^+^ by BLAST search^57^ using the criteria of nucleotide sequences >3 kb matched with 97% identity and a gap length of less than 100 bp, and then the unmapped long reads were obtained. For the individual mating types, RNA-seq data derived from the gametes and the gametophytes before gametogenesis were assembled into isotigs using Newbler (Roche Applied Science, Penzberg, Germany) with default parameters, and these isotigs were used as gene models. The isotigs of a mating type were mapped on the scaffolds of the same mating type. The mt^−^ unmapped long reads on the above mt^+^ long scaffolds were used as query sequences against a database generated from the mt^−^ scaffolds using BLASTN, and a pool of long reads with e-values of >1 × 10^−100^ was selected. From the selected long reads, MTS reads were defined using the following criteria: 1) identity with a region overlapping the read was less than 80%, 2) the read contained the gene model(s), and 3) length >1 kb. An equivalent analysis of the mt^+^ long reads was also performed. Finally, 241 and 320 sites were identified for mt^−^ and mt^+^, respectively. A summary of the MTS reads is shown in Supplementary Table 2.

#### Identification of MTS genomic scaffolds

To identify genomic regions in which successively mapped MTS reads were present, “moving sums” were calculated. The length of a read among the MTS reads on a scaffold and lengths of n − 1 reads toward the 3′ end from the first read were summed (*l*, vertical axis in Supplementary Fig. 3) and defined as the “moving sum” unit. From a read at the 5′ end of a scaffold to its 3′ end, the same calculations of moving sums were performed. In addition, the distance between the position at the 5′ end of the first read of a moving sum and the position at the 3′ end of its last read, which were n reads, was calculated (*L*, horizontal axis in Supplementary Fig. 3). All moving sums for n = 5, 10, and 15 were calculated. The *l/L* ratio was used as an index of the extent to which the MTS reads were successive on a scaffold that is part of the *U*. *partita* genome. If the moving sum is completely successive, the ratio will be >1. MTSs that met the following criteria were subjected to further analysis: (1) *l/L* was >0.1, (2) *l* was >50 kb in n = 15, and (3) *L* was >1 Mb. Scaffold 632 from mt^−^ and Scaffold 4 and Scaffold 898 from mt^+^ were identified as containing highly successive MTS reads (HMTS scaffolds).

#### Comparison of MTS genomic scaffolds from mt^−^ and mt^+^ genomes

The data from short reads, MTS reads, Cufflinks gene models (not the same as the models from isotigs: see RNA-seq analysis for the generation of these gene models), and RNA-seq reads from gametes and gametophytes were mapped onto the scaffolds and visualized using the GBrowse genome browser^58^. The HMTS scaffolds were used as query sequences against the database for the opposite mating type using LAST (long-sequence alignment software) with default parameters^59^, and their counterpart scaffolds were identified. This process was performed reciprocally, and three scaffolds for mt^−^ (#632, #629, and #1214) and four scaffolds for mt^+^ (#4, #898, #462, and #469) were identified. These scaffolds were aligned and visualized as a dot plot using a script in the LAST software with default parameters. Finally, the mt^−^ and mt^+^ HMTS scaffolds were estimated as complementary scaffolds, and the sums of the HMTS scaffolds and adjacent scaffolds for mt^−^ and mt^+^ were 7.19 and 7.33 Mb, respectively.

#### Comparison and visualization of the MT locus

The regions containing HMTS reads were ~1.0 and ~1.5 Mb, respectively. RNA-seq data of gametes and gametophytes in the individual mating types were merged and assembled using Newbler, and CDSs were generated automatically. These isotigs, were mapped on the genomic assemblies of both mating types, and genes contained in these regions were identified from the isotig gene models. In total, 84 and 95 genes with splicing variants were identified for mt^−^ and mt^+^, respectively (Supplementary Tables 3 and 4). Clustering analyses using the DNACLUST package^60^ were performed for the genes in the HMTS genomic regions in mt^−^ and mt^+^; after manual correction of the clusters, they were classified into 46 and 67 clusters that were defined as splicing variants transcribed from individual loci. These genes were compared by using reciprocal BLASTX analyses^61^ with e-values of >1 × 10^−3^, and 23 were identified as gametologs (Supplementary Table 5). Representative genes were selected and their positional data in the scaffolds were visualized using the ggplot2 (1.0.0) and ggbio (1.14.0)^62^ packages in R (3.1.3). The resulting data were modified using drawing software. The identified regions in the mt^−^ and mt^+^ scaffolds were termed the MT loci. The nucleotide sequences of the MT loci were submitted to the DNA Data Bank of Japan (DDBJ; accession numbers: LC091542 for the mt^+^ MT locus; and LC091540 and LC091540 for mt^−^ MT loci in scaffolds 4 and 898, respectively). The nucleotide sequences of the MT locus genes for RNA-seq analysis were submitted to DDBJ, and the accession numbers are shown in Supplementary Tables 3 and 4.

#### Segregation analysis

From MGEC-5 (mt^−^) and MGEC-2 (mt^−^) gametophytes, gametes were induced and mixed. Then, mated zygotes were gathered together by negative phototaxis. After cultivation for 3 weeks, some sporophytes were transferred to MGEC-5 or MGEC-2 culture flasks, from which zoospores were induced by the same method as for the gametogenesis. Microscopy was used to determine whether the zoospores had four flagella, which are different from gametes with two flagella. Zoospores that showed exactly four flagella were cultured in 1-L flasks for 3 weeks. Approximately 100 small gametophytes of MGEC-5/MGEC-2 progeny were transferred into respective 1-L flasks and cultured for 3 weeks. Before checking the mating types, small pieces of thalli were collected, frozen in liquid nitrogen and stored at −80 °C until the extraction of genomic DNA. Gametes were induced from approximately 50 healthily developed gametophytes. MGEC-2 (mt^−^) and MGEC-1 (mt^+^) were used as testers. After mixing the gametes of MGEC-5/MGEC-2 progeny with mt^−^ or mt^+^ testers, mating was checked by negative phototaxis and the observation of zygotes. Finally, the mating types of a total of 16 MGEC-5/MGEC-2 progeny were determined.

#### Identification of repeats

To identify repetitive sequences, we used RepeatMasker (ver. 4.05) and RepBase (20140131) with RepeatMasker in polymorphism mode (Open-4.0. 2013–2015, http://www.repeatmasker.org). From all mt^−^ genome assemblies, in total, 92 kb of repeats containing 1,126 transposable elements, 223 small RNAs, and one satellite were identified. For all mt^+^ genome assemblies, in total, 106 kb of repeats containing 1,147 transposable elements, 173 small RNAs, and one simple repeat were identified. The data of the mt^−^ scaffolds (#632, #629, and #1214) and the mt^+^ scaffolds (#4, #898, #462, and #469) were extracted and visualized using R/ggbio.

### Molecular evolution analysis

#### Phylogenetic analysis

To construct phylogenetic trees, an MT locus gene (PRA1) and a gene (GTBP1) in the flanking region of the MT locus (homologous genes) were isolated from *Ulva* spp. (Supplementary Table 7). The CDS regions of the two genes from the examined species were amplified using degenerate primers (mt^−^ *PRA1m*/mt^+^ *PRA1f*, 5′-TTCATTGCYGTTCAAGCTACWAC-3′ and 5′-AACAAGCTCWCCRTCTTTCTCCCA-3′; G-strand telomere-binding protein 1 (*GTBP1*), 5′-TGGCGCACATCATGGCAAGATT-3′ and 5′-CAGCCCCACTGATCGAGCTTCAC-3′). The PCR program for *PRA1* and *GTBP1* consisted of one initial denaturation step for 2 min at 94 °C, followed by 45 cycles of denaturation for 30 s at 94 °C, annealing for 30 s at 50 °C, and extension for 40 s at 68 °C. Sequences were aligned using Muscle in MEGA6^63^. Model tests for each analysis were performed using KAKUSAN 4.0^64^. The best fit models for maximum-likelihood analysis were GTR + G for mt^−^ *PRA1m*/mt^+^ *PRA1p* and J2 + G for *GTBP1*, based on the Akaike information criterion (AIC). Phylogenetic analyses were performed using the maximum-likelihood method in TREEFINDER^65^. Bootstrap values^66^ were obtained from analyses of 100 pseudoreplicates. The nucleotide sequences of the genes homologous to mt^−^ *PRA1m*/mt^+^ *PRA1p* and GTBP1 were submitted to DDBJ, and the accession numbers are shown in Supplementary Table 7.

Using the *C*. *reinhardtii* RWP-RK domain-containing proteins defined by Chardin *et al*.^45^ as queries, RWP-RK domain-containing proteins were retrieved by BLAST analysis from data sets in Phytozome 11 (http://phytozome.jgi.doe.gov/pz/portal.html) for *C*. *reinhardtii* (ver. 5.5), *Volvox carteri* (ver. 2.1), *Coccomyxa subellipsoidea* (ver. 2.0), and *Micromonas pusilla* (ver. 3.0), and from NCBI for *Gonium pectorale* ^38^. *MID*s for *Chlamydomonas* and *Volvox* were retrieved from NCBI^9,13^. *U*. *partita* autosomal genes encoding an RWP-RK domain-containing protein were identified with BLASTX using all *Chlamydomonas* RWP-RP domain-containing proteins as queries, similar to the description above, against the RNA-seq assembly database. The resulting data set served as input for a conserved motif analysis performed using MEME (http://meme.sdsc.edu/meme/meme.html), and five conserved motifs were identified. The five motifs were combined and used for molecular phylogenetic analyses. Phylogenetic analyses were performed using the maximum-likelihood method in MEGA6. Model tests for the analysis were also performed using MEGA6. The best-fit model, based on AIC, for the RWP-RK domain-containing proteins was JTT + G. Bootstrap values were obtained from analyses of 100 pseudoreplicates.

#### Calculation of synonymous and non-synonymous substitution rates

From mRNA assembly data, CDSs of gametologs were extracted, and the deduced amino acid sequences were checked manually with BLAST analyses. If the amino acid sequences were not similar between gametologs, the full-length assembly sequences were analyzed with ORF finder (http://www.ncbi.nlm.nih.gov/gorf/gorf.html) and an appropriate frame was identified. After manual checking, two gametologs were found to contain no CDSs that could form pairs with their counterparts. Pairs of CDSs for the individual gametologs were aligned using the ParaAT package^67^, and the alignments obtained were used to calculate the synonymous and non-synonymous substitution rates using the maximum-likelihood and approximation methods with nucleotide substitution models in the KaKs_Calculator 2.0 package^68,69^. For comparison with the substitution rate in *U*. *partita*, CDSs of gametologs in *C*. *reinhardtii* and *V*. *carteri* ^9,13^ were obtained from the NCBI database (Supplementary Table 8). The data were plotted using ggplot2 for R.

#### Calculation of codon usage

CDSs of all mRNAs of mt^−^ and mt^+^ were extracted using a custom-made Perl script, and codons in individual CDSs were counted using the cusp command of EMBOSS (ver. 6.6.0.0). The results were merged using a custom-made Python script. From these data, the MT locus genes and autosomal genes were separated, and Pearson’s product-moment correlations and p-values for the sums of individual codons of all autosomal genes and the MT locus genes were determined using the R default “cor” command. The correlation between all autosomal mt^−^ and mt^+^ genes was 0.98 (p = 2.2 × 10^−16^).

#### Amplification of MT locus genes

DNA was extracted from six *U*. *partita* strains using the CicaGeneus DNA Extraction Reagent DNA kit (Kanto Chemical, Tokyo, Japan). The mating types are given in Supplementary Table 7. To amplify DNA fragments of individual genes, the Kapa Taq PCR kit (Kapa Biosystems) was used in accordance with the manufacturer’s protocol. The PCR program for the amplification of each gene consisted of an initial denaturation step of 3 min at 95 °C, followed by 45 cycles of denaturation for 15 s at 95 °C, annealing for 15 s at 60 °C, and extension for 90 s at 72 °C. Primer sets are shown in Supplementary Table 15. The amplified DNA fragments were separated by electrophoresis and visualized by ethidium bromide staining.

#### RNA-seq analysis

Triplicate RNA-seq data from gametophytes, gametophytes after the induction of gametogenesis (24 and 48 h), and gametes from mt^−^ and mt^+^ were obtained using an Illumina HiSeq. 2500. To compare the transcripts from the mt^−^ and mt^+^ strains, the gamete and gametophyte data were merged and mapped on the mt^−^ scaffolds and gene models (Cufflinks gene models) generated using a TopHat-Cufflinks pipeline, TopHat2 (2.0.12)/Bowtie2 (2.2.3)/Cufflinks (2.2.1)^70–72^. To compare gene expression at the MT locus during gametogenesis between the two mating types, mRNA gene models were used for the analysis using Cuffmerge from the Cufflinks results of individual genes of fragments per kilobase of exon per million fragments of mapped reads (FPKM) values, and the data were aggregated. The sum of FPKM values of all splicing variants at a locus was used to compare gametolog expression between the two mating types. Mean FPKM values were normalized relative to the maximum values, and clustering analysis was performed by the *k*-means method. One-way ANOVA and the Wilcoxon rank-sum test were performed for the expression data of each MT locus gene during gametogenesis. All statistical analyses were performed using R.

## Electronic supplementary material

Supplementary Text, Figures and Tables

Supplementary Tables
