# Supplementary material for: Genomic structure and evolution of the mating type locus in the green seaweed Ulva partita
Source: Sci Rep. 2017 Sep 15;7:11679. doi: 10.1038/s41598-017-11677-0 (PMC5601483; doi:10.1038/s41598-017-11677-0)
Supplement: Supplementary file 1 — Supplementary Text, Figures and Tables [file 41598_2017_11677_MOESM1_ESM.pdf]

Title

Genomic structure and evolution of the mating type locus in the green seaweed *Ulva partita*

Authors

Tomokazu Yamazaki, Kensuke Ichihara, Ryogo Suzuki, Kenshiro Oshima, Shinichi Miyamura, Kazuyoshi Kuwano, Atsushi Toyoda, Yutaka Suzuki, Sumio Sugano, Masahira Hattori and Shigeyuki Kawano

Supplementary Text

Identification of mating type-specific regions

Some previous studies aiming to identify sex chromosomes were based on mapping coverage of short reads from the diploid genome of one sex on another diploid genome of the opposite sex, and the mapped reads per site on the sex chromosome (Y or W) were half for autosomes, leading to identification of the sex chromosomes [1,2]. However, our macroalgal genomic DNA samples were obtained from the haploid phase, so the genomic data obtained consisted of only haploid genome sequences. Thus, a strategy was required to identify the MT locus from whole-genome sequences of mt^{-} and mt^{+} strains. In this study, we used a single-molecule sequencing technology, the PacBio system, in which the individual sequence reads obtained are very long, as opposed to the short- to medium-length reads yielded by sequencing systems such as the Illumina and Roche 454 platforms. Comparison of whole-genome sequences between mt^{-} and mt^{+} is difficult, because the sequence data obtained from scaffolds after assembly of the raw data are generally incomplete, and it is unclear how the scaffolds for one mating type are associated with those for the other mating type and where individual loci on scaffolds are located in each genome. Therefore, we first attempted a mapping-based strategy to detect large overall differences between the two genomes using unassembled PacBio long reads and the scaffolds assembled with them, and to identify mating type-specific PacBio reads. Regions that differ significantly between the genomes, such as the mating loci and SDRs, are potentially candidate MT loci. If the identified regions are integrated viruses, major histocompatibility complex (MHC)-like incompatibility regions, or genomic islands, they are expected to contain many transposable elements, many genes within the same family, or horizontally transferred

genes, allowing them to be ruled out as potential candidates [3-5]. To exclude these divergent regions, we applied a second strategy in which the degree of adjacency of the mating type-specific PacBio reads was used as an index.

First, the PacBio long reads (1.7×10^6 reads and 12.0 Gb for mt$^-$ and 2.7×10^6 reads and 16.6 Gb for mt$^+$) from both mating type genomes were assembled after correction of the misreading of sequence data with Illumina short reads (Supplementary Table 1). The assemblies of these data generated 851 and 1,385 scaffolds for the two mating types; the sums of the scaffolds, which are thought to reflect the genome sizes, were 110.2 and 116.7 Mb, respectively. Using BLASTN, the corrected long reads from one mating type were compared with the total scaffolds of the opposite mating type, and long reads with low similarity (< 80%) were selected. These low-similarity long reads were thought to be mating type-specific (MTS) long reads. After this selection, two sets of MTS long reads were obtained, numbering 214 for mt$^-$ and 320 for mt$^+$ (Supplementary Table 2). To determine the distribution of the MTS long reads on the genome, they were mapped on the scaffolds assembled from PacBio reads of the individual mating types (Supplementary Fig. 1).

The results showed that the MTS long reads were not distributed on a particular scaffold but over multiple scaffolds, suggesting that these MTS long reads may contain highly polymorphic sites and that these regions are scattered throughout the genome. The well-known mating type (MT) locus, the SDR of UV chromosomes, and Y/W chromosome-specific region are genomic regions that differ markedly in structure between individual mating types or sexes. If a region of this kind is present in the genome, it is likely a site where numerous MTS long reads are located close together and, more specifically, are adjacent to each other. To extract the MTS long reads that

exhibited a high level of adjacency in the genomes, the “moving sum” of PacBio long reads was used as an index, where lengths of 5, 10, or 15 successive MTS long reads were summed; the same calculations were performed for all successive reads (see Materials & Methods for a detailed definition of the moving sum). In addition, the distance between the two distal ends of a set of successive reads was calculated using the positions mapped on the scaffolds of the genome. The same calculations were performed for all MTS long reads. The sums of the MTS read lengths and the distances between the two ends of each set of successive reads were plotted (Supplementary Fig. 2). The more a set of MTS long reads exhibited a highly adjacent arrangement, the more the sum of reads approached the full length of the genome and some successive read sets showed the same relationships. Although the sum of their lengths did not correspond to the distance between the two ends for any set of reads, some sets showed a ratio > 0.1 and < 0.5 (Supplementary Fig. 3A-C). Among these read sets, those mapped on mt^{-} scaffold 632 showed significantly homogeneous ratios.

References

1 Carvalho, A. B. & Clark, A. G. Efficient identification of Y chromosome sequences in the human and Drosophila genomes. *Genome Res* **23**, 1894-1907 (2013).

2 Chen, S. L. *et al.* Whole-genome sequence of a flatfish provides insights into ZW sex chromosome evolution and adaptation to a benthic lifestyle. *Nat Genet* **46**, 253-260 (2014).

3 de Bakker, P. I. W. *et al.* A high-resolution HLA and SNP haplotype map for disease association studies in the extended human MHC. *Nat Genet* **38**, 1166-1172 (2006).

4 Juhas, M. *et al.* Genomic islands: tools of bacterial horizontal gene transfer and evolution. *Fems Microbiol. Rev.* **33**, 376-393 (2009).

5 Minot, S. *et al.* Rapid evolution of the human gut virome. *Proceedings of the National Academy of Sciences of the United States of America* **110**, 12450-12455 (2013).

Supplementary Tables

Supplementary Table 1. Summary of sequence data

Supplementary Table 2. Summary of mating type-specific PacBio reads

Supplementary Table 3. MT locus genes in the mt^{-} strain

Supplementary Table 4. MT locus genes in the mt^{+} strain

Supplementary Table 5. Gametologs in the MT locus

Supplementary Table 6. Segregation of the *U. partita* MT locus genes among progeny of zoospores generated by the mating of two isolates

Supplementary Table 7. List of specimens used in this study

Supplementary Table 8. Genes in the MT locus of *U. partita* and other green algae

Supplementary Table 9. dN and dS values of gametologs in the MT locus of *U. partita* and other green algae

Supplementary Table 10. Distance between two points for dN and dS values of *U. partita* gametologs

Supplementary Table 11. Distance between two points for dN and dS values of *C. reinhardtii* gametologs

Supplementary Table 12. Distance between two points for dN and dS values of *V. carteri* gametologs

Supplementary Table 13. Averages of dN, dS, and dN/dS values for three species

Supplementary Table 14. Numbers of codons in individual MT locus genes, summed numbers of all autosomal genes, and results of correlation analysis

Supplementary Table 15. Expression levels of MT locus genes during gametogenesis and results of statistical testing

Supplementary Table 16. Primer sets for MT locus genes

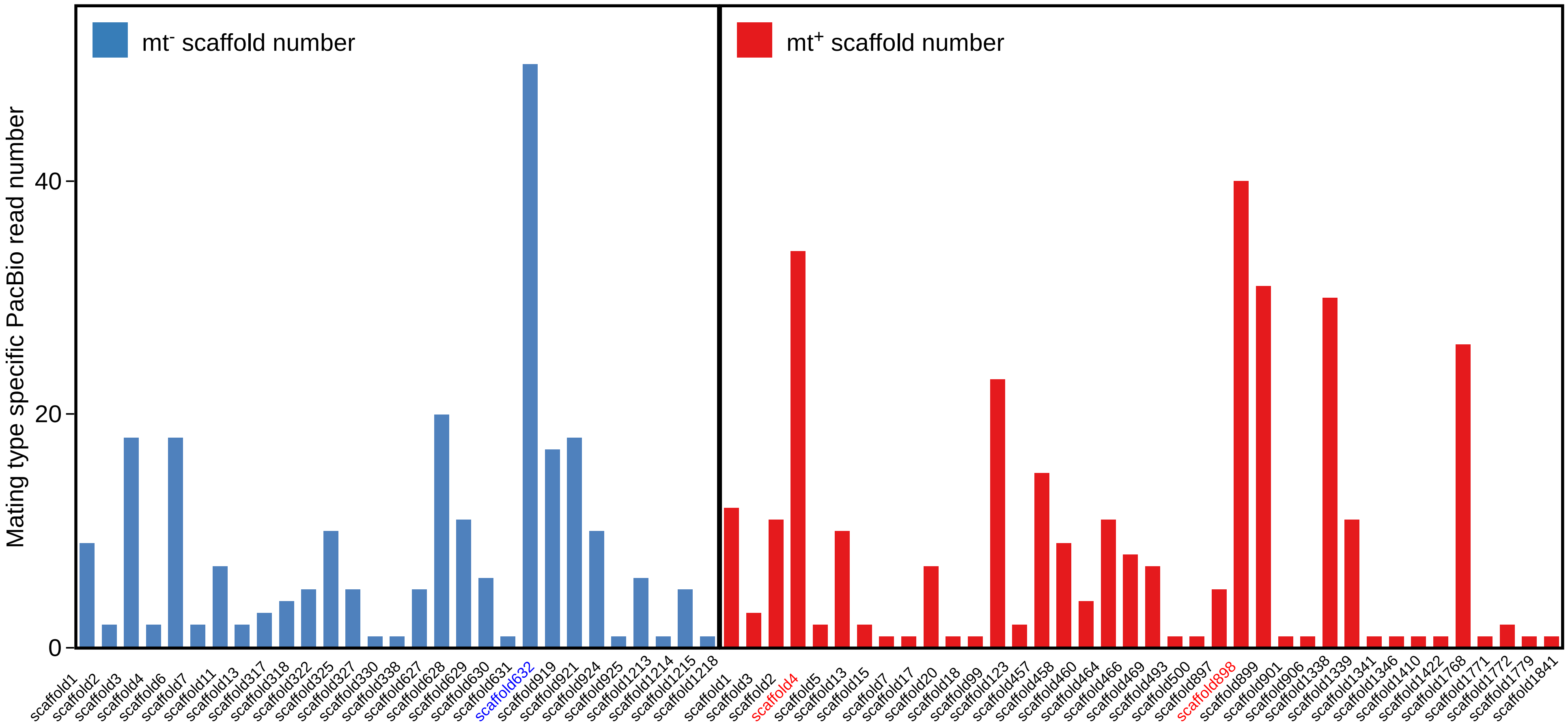

mt- scaffold number
mt+ scaffold number
Mating type specific PacBio read number
0
20
40
scaffold1
scaffold2
scaffold3
scaffold4
scaffold6
scaffold7
scaffold11
scaffold13
scaffold317
scaffold318
scaffold322
scaffold325
scaffold327
scaffold330
scaffold338
scaffold627
scaffold628
scaffold629
scaffold630
scaffold631
scaffold632
scaffold919
scaffold921
scaffold924
scaffold925
scaffold1213
scaffold1214
scaffold1215
scaffold1218
scaffold1
scaffold3
scaffold2
scaffold4
scaffold5
scaffold13
scaffold15
scaffold7
scaffold17
scaffold20
scaffold18
scaffold99
scaffold123
scaffold457
scaffold458
scaffold460
scaffold464
scaffold466
scaffold469
scaffold493
scaffold500
scaffold897
scaffold898
scaffold899
scaffold901
scaffold906
scaffold1338
scaffold1339
scaffold1341
scaffold1346
scaffold1410
scaffold1422
scaffold1768
scaffold1771
scaffold1772
scaffold1779
scaffold1841

Supplementary Figure 1. **Distribution of mating type-specific reads on scaffolds**. Mating type-specific PacBio reads were mapped on scaffolds of the same mating type, enumerated, and plotted as bar graphs. The indexes of the MT locus scaffolds are in blue for mt^{-} and in red for mt^{+}.

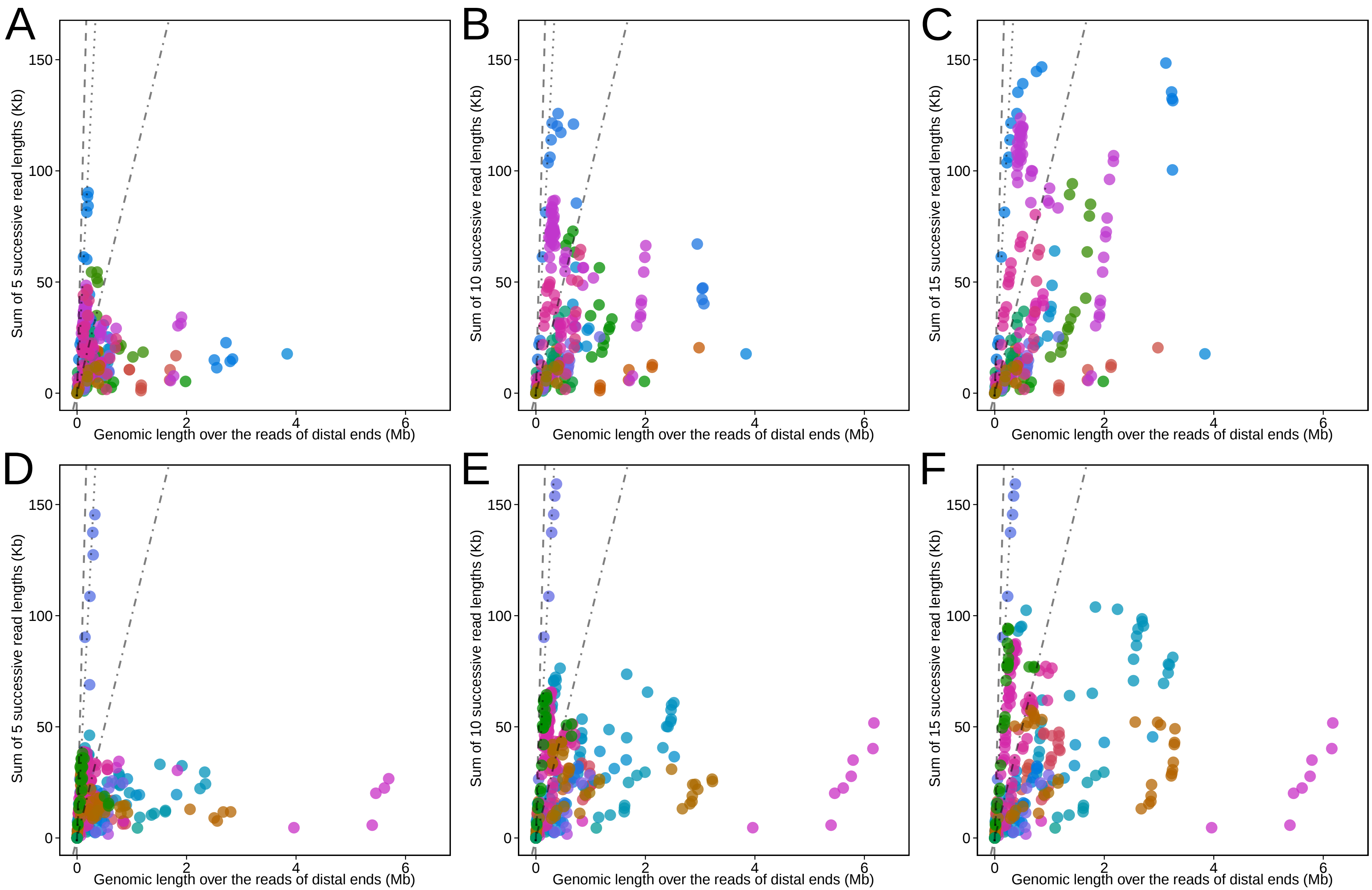
A
B
C
D
E
F
Sum of 5 successive read lengths (Kb)
Sum of 10 successive read lengths (Kb)
Sum of 15 successive read lengths (Kb)
Genomic length over the reads of distal ends (Mb)
0
50
100
150
0
2
4
6

Supplementary Figure 2. **Moving sum of mating type-specific PacBio reads and the genomic distance from the start to the end point of calculation**. The sums of the lengths of successive mating type-specific PacBio reads and the genomic length from the first read to the end read were calculated. (A-C) mt^{-}-specific reads. (D-F) mt^{+}-specific reads. (A) and (D) Moving sums of five reads. (B) and (E) Moving sums of 10 reads. (C) and (F) Moving sums of 15 reads. Lines show the ratios of the sum of the sequential read length per genomic length. Dashed line, 1, Dotted line, 0.5, Dashed and dotted line, 0.1. The reads plotted under the dashed and dotted lines are low successive reads. The colors indicate that the reads were derived from the corresponding scaffold.

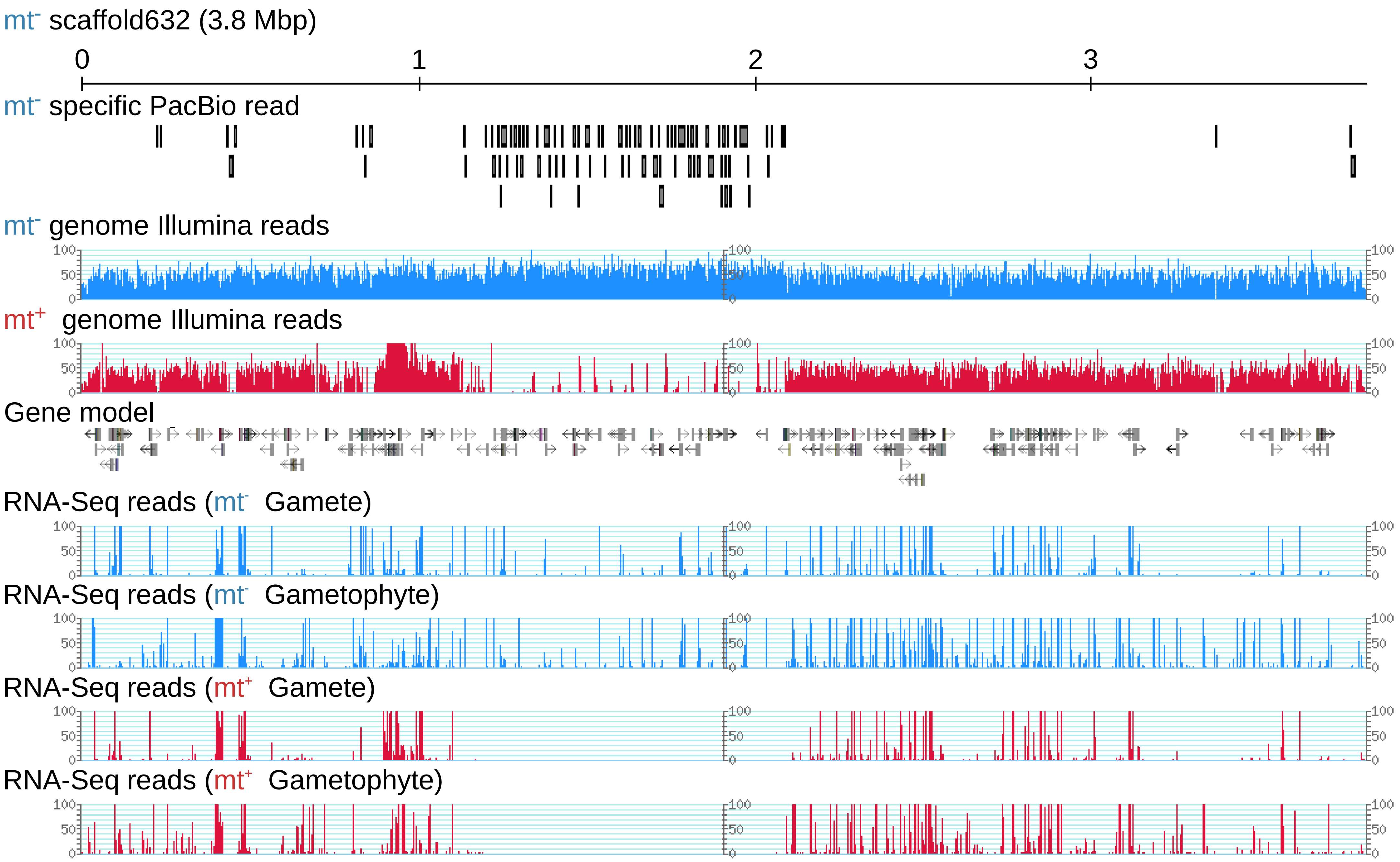

mt- scaffold632 (3.8 Mbp)
0
1
2
3
mt- specific PacBio read
mt- genome Illumina reads
mt+ genome Illumina reads
Gene model
RNA-Seq reads (mt- Gamete)
RNA-Seq reads (mt- Gametophyte)
RNA-Seq reads (mt+ Gamete)
RNA-Seq reads (mt+ Gametophyte)
100
50
0

Supplementary Figure 3. **Mapping of gene models derived from mating type-specific PacBio reads, DNA-Seq Illumina reads, and RNA-seq Illumina reads on mt^{-} scaffold 632**. The identified mt^{+}-specific scaffold 632 is shown. The upper lane is a ruler for position within the whole scaffold. The length of scaffold 632 is approximately 3.8 Mb. The second lane shows the positions of mating type-specific PacBio reads mapped onto this scaffold. The third and fourth lanes show the densities of reads derived from mt^{-} (blue) and mt^{+} (red) genomes by Illumina sequencing. The fifth lane shows the positions of predicted genes from RNA-seq assemblies. Lanes 6 to 9 are mapped fragments derived from RNA-seq assemblies on this scaffold. The RNA-seq assemblies are derived from gametes or gametophytes of mt^{-} (blue, lanes 6 and 7) and mt^{+} (red, lanes 8 and 9) strains. Each lane was visualized using GBrowse.

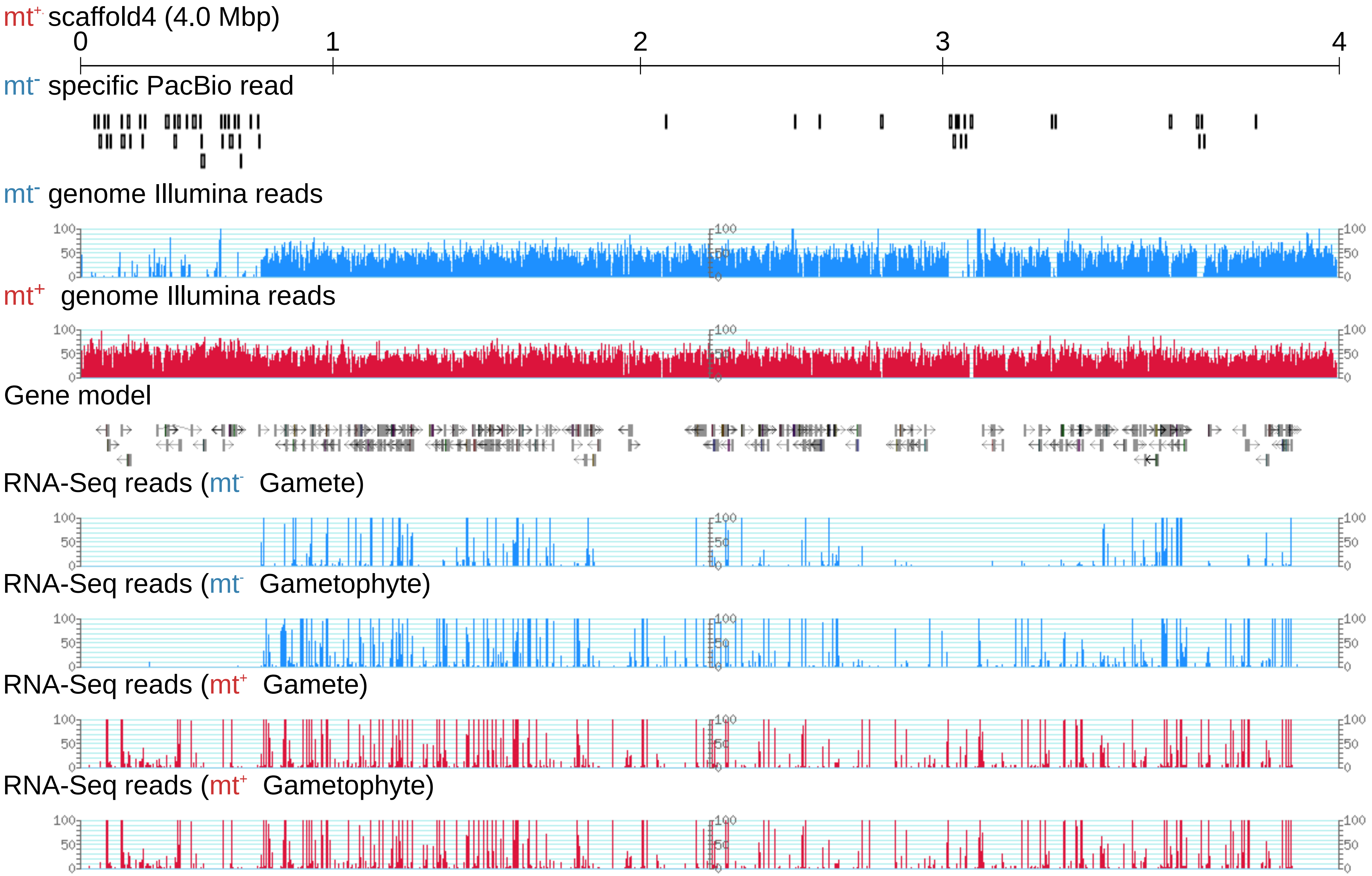

mt+ scaffold4 (4.0 Mbp)
0
1
2
3
4
mt- specific PacBio read
mt- genome Illumina reads
100
50
0
mt+ genome Illumina reads
Gene model
RNA-Seq reads (mt- Gamete)
RNA-Seq reads (mt- Gametophyte)
RNA-Seq reads (mt+ Gamete)
RNA-Seq reads (mt+ Gametophyte)

Supplementary Figure 4. **Mapping of gene models derived from mating type-specific PacBio reads, DNA-Seq Illumina reads, and RNA-seq Illumina reads on mt^{+} scaffold 898**. The identified mt^{+}-specific scaffold 898 is shown. The upper lane is a ruler for position within the whole scaffold. The length of scaffold 898 is approximately 0.9 Mb. The second lane shows the positions of mating type-specific PacBio reads mapped onto this scaffold. The third and fourth lanes show the densities of reads derived from mt$^{−}$ (blue) and mt^{+} (red) genomes by Illumina sequencing. The fifth lane shows the positions of predicted genes from RNA-seq assemblies. Lanes 6 to 9 are mapped fragments derived from RNA-seq assemblies on this scaffold. The RNA-seq assemblies are derived from gametes or gametophytes of mt$^{−}$ (blue, lanes 6 and 7) and mt^{+} (red, lanes 8 and 9) strains. Each lane was visualized using GBrowse.

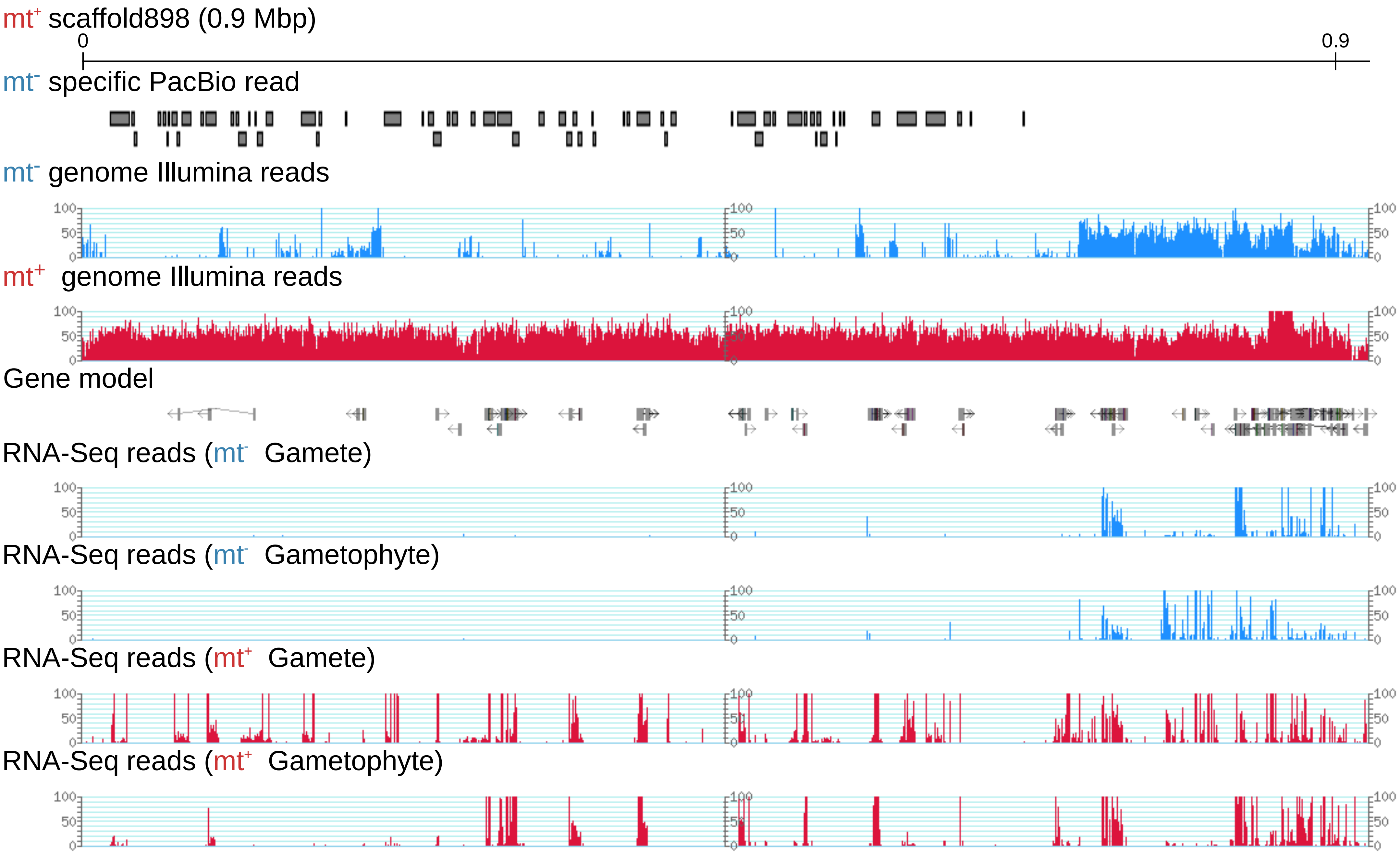
mt+ scaffold898 (0.9 Mbp)
0
0.9
mt- specific PacBio read
mt- genome Illumina reads
100
50
0
mt+ genome Illumina reads
Gene model
RNA-Seq reads (mt- Gamete)
RNA-Seq reads (mt- Gametophyte)
RNA-Seq reads (mt+ Gamete)
RNA-Seq reads (mt+ Gametophyte)

Supplementary Figure 5. **Mapping of gene models derived from mating type-specific PacBio reads, DNA-Seq Illumina reads, and RNA-seq Illumina reads on mt^{+} scaffold 4**. The identified mt^{+}-specific scaffold 4 is shown. The upper lane is a ruler for whole scaffold positions. The length of scaffold 4 is approximately 4.0 Mb. The second lane shows the positions of mating type-specific PacBio reads mapped onto this scaffold. The third and fourth lanes show the densities of reads derived from mt^{-} (blue) and mt^{+} (red) genomes by Illumina sequencing. The fifth lane shows the positions of predicted genes based on RNA-seq assemblies. Lanes 6 to 9 are mapped fragments derived from RNA-seq assemblies on this scaffold. The RNA-seq assemblies are derived from gametes or gametophytes of mt^{-} (blue, lanes 6 and 7) and mt^{+} (red, lanes 8 and 9) strains. Each lane was visualized using GBrowse.

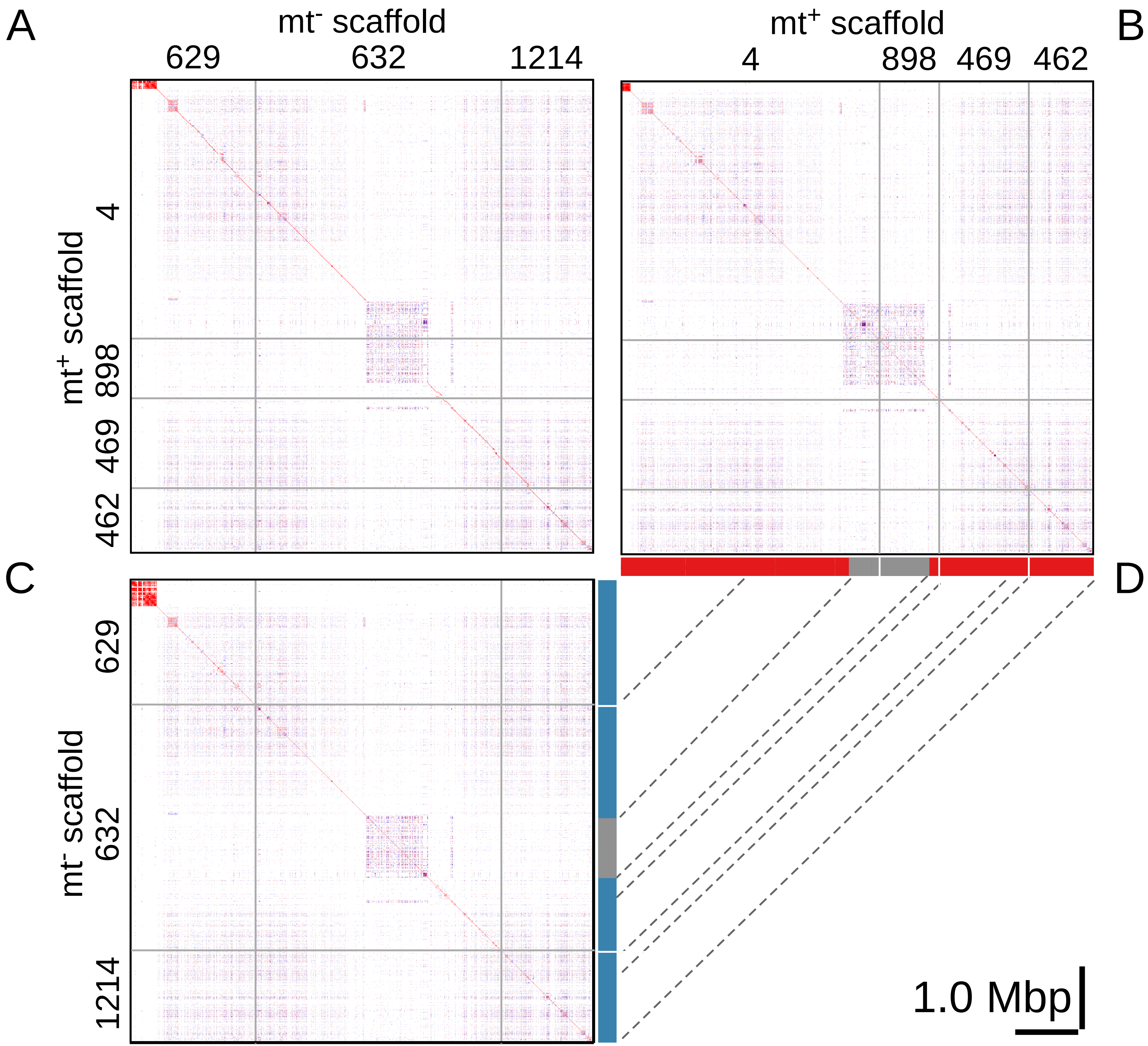
A
mt- scaffold
629
632
1214
mt+ scaffold
4
898
469
462
B
mt+ scaffold
4
898
469
462
C
629
mt- scaffold
632
1214
D
1.0 Mbp

Supplementary Figure 6. **Dot plot of nucleotide sequences for MT and surrounding scaffolds**. Mating type-specific scaffolds and adjacent scaffolds were compared by dot-plot analysis using the LAST software. Adjacent scaffolds were identified by a reciprocal BLAST search. (A) Comparison of mt^{-} and mt^{+} scaffolds. (B) Comparison of mt^{+} scaffolds. (C) Comparison of mt^{-} scaffolds. (D) Schematics of the scaffolds for individual mating types. Bar indicates 1 Mb.

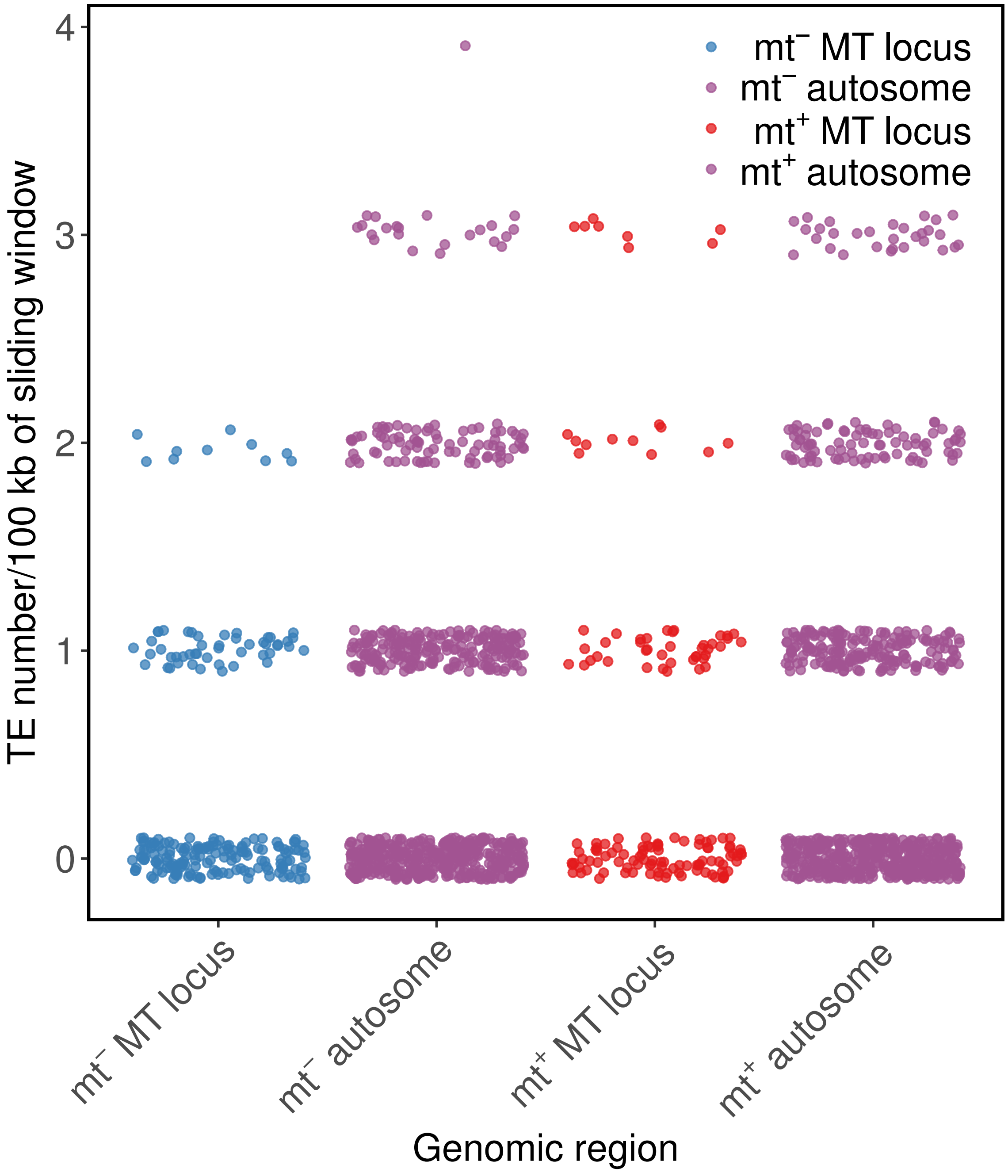

mt⁻ MT locus
mt⁻ autosome
mt⁺ MT locus
mt⁺ autosome
TE number/100 kb of sliding window
0
1
2
3
4
mt⁻ MT locus
mt⁻ autosome
mt⁺ MT locus
mt⁺ autosome
Genomic region

Supplementary Figure 7. **Numbers of predicted transposable elements in the MT locus and neighborhoods**. The numbers of predicted transposable elements were calculated separately for each 100 kbp in the MT locus and neighbor regions. The data were plotted by scatterplot with jittering. Blue, mt^{-}; red, mt^{+}; purple, neighbor regions.

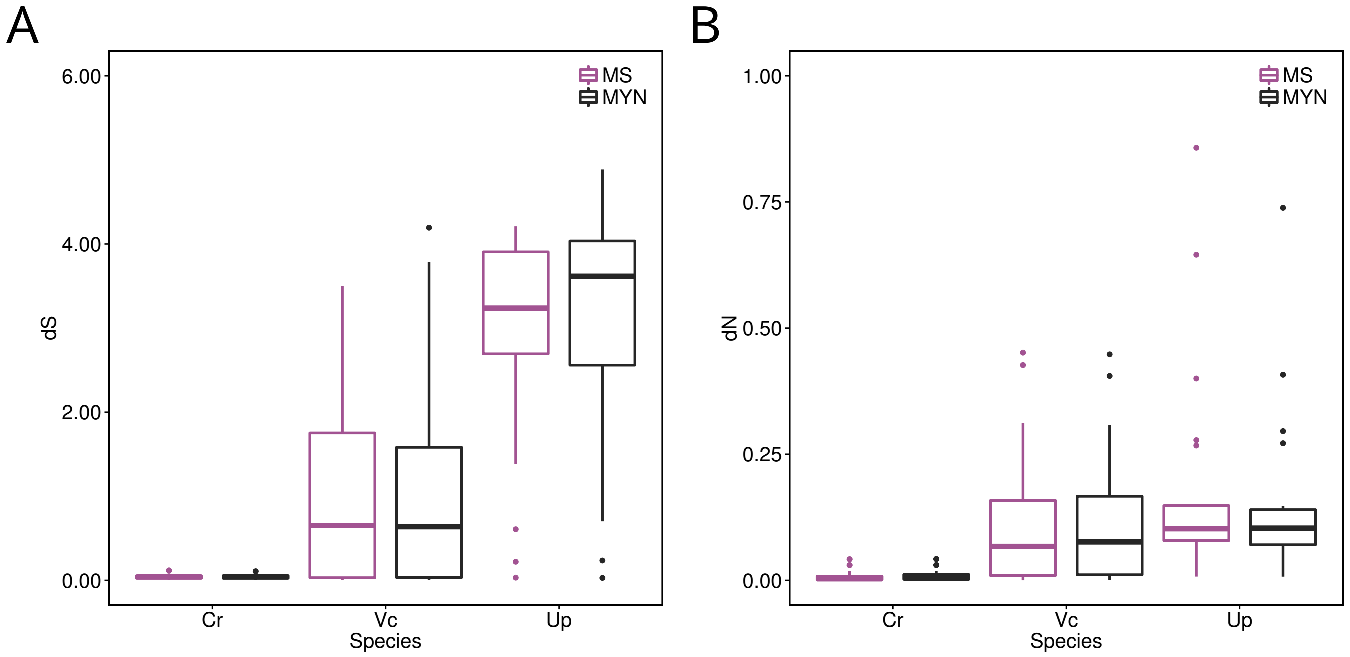
A
B
MS
MYN
dS
dN
6.00
4.00
2.00
0.00
1.00
0.75
0.50
0.25
Cr
Vc
Up
Species

Supplementary Figure 8. **Box-whisker plots of dS and dN**. Box-whisker plots show the distributions of the dS (A) and dN (B) values for gametologs in three species. The dS and dN were estimated by the model selection (MS) method. MYN, modified YN method. Cr, *C. reinhardtii*, Vc, *Volvox carteri*, Up, *Ulva partita*.

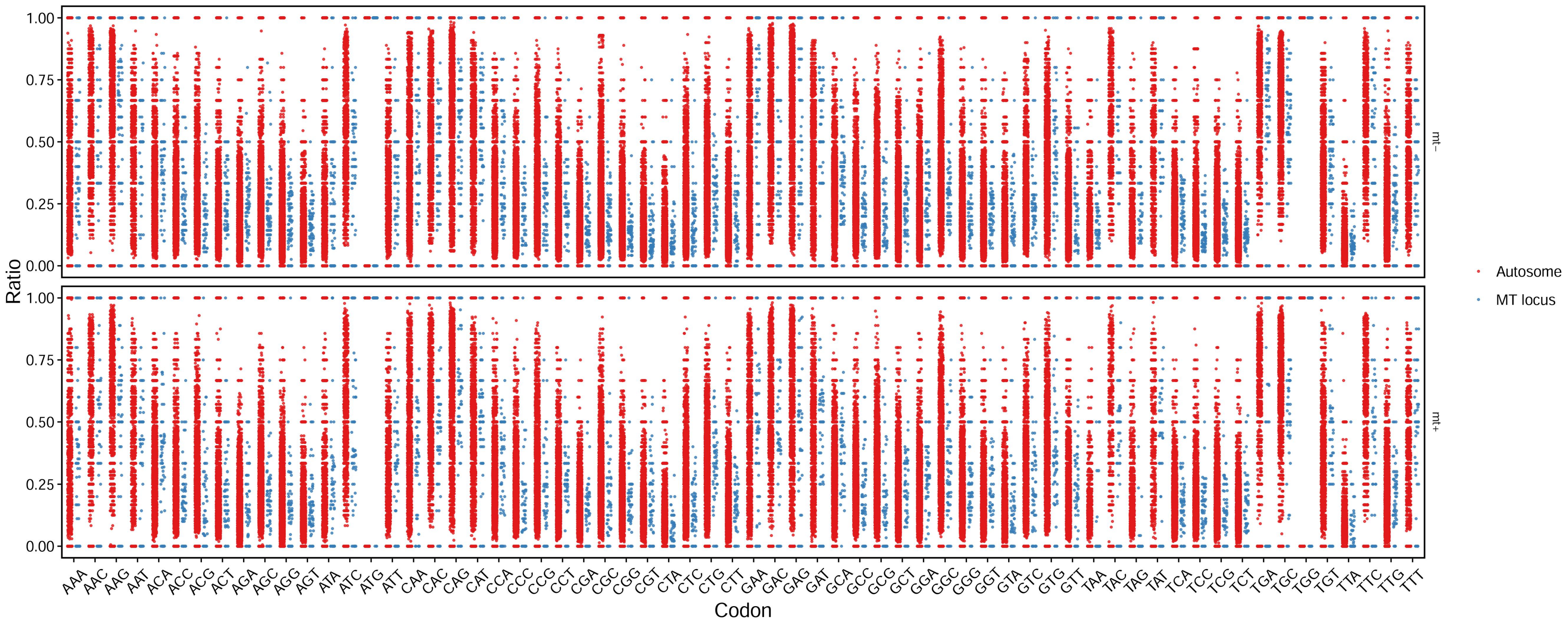

Ratio
Codon
mt−
mt+
Autosome
MT locus
1.00
0.75
0.50
0.25
0.00
AAA AAC AAG AAT ACA ACC ACG ACT AGA AGC AGG AGT ATA ATC ATG ATT CAA CAC CAG CAT CCA CCC CCG CCT CGA CGC CGG CGT CTA CTC CTG CTT GAA GAC GAG GAT GCA GCC GCG GCT GGA GGC GGG GGT GTA GTC GTG GTT TAA TAC TAG TAT TCA TCC TCG TCT TGA TGC TGG TGT TTA TTC TTG TTT

Supplementary Figure 9. **Codon usage in autosomal and MT locus genes**. Codon usage in autosomal genes and MT locus genes was quantified. From these data, the ratios of individual codons of individual genes were calculated and plotted. Therefore, each dot indicates the codon ratio of a gene. Upper panel, results for mt^{-} genes. Lower panel, results for mt^{+} genes. Red dots, codon usage of autosomal genes. Blue dots, codon usage of MT locus genes. Index shows codons.

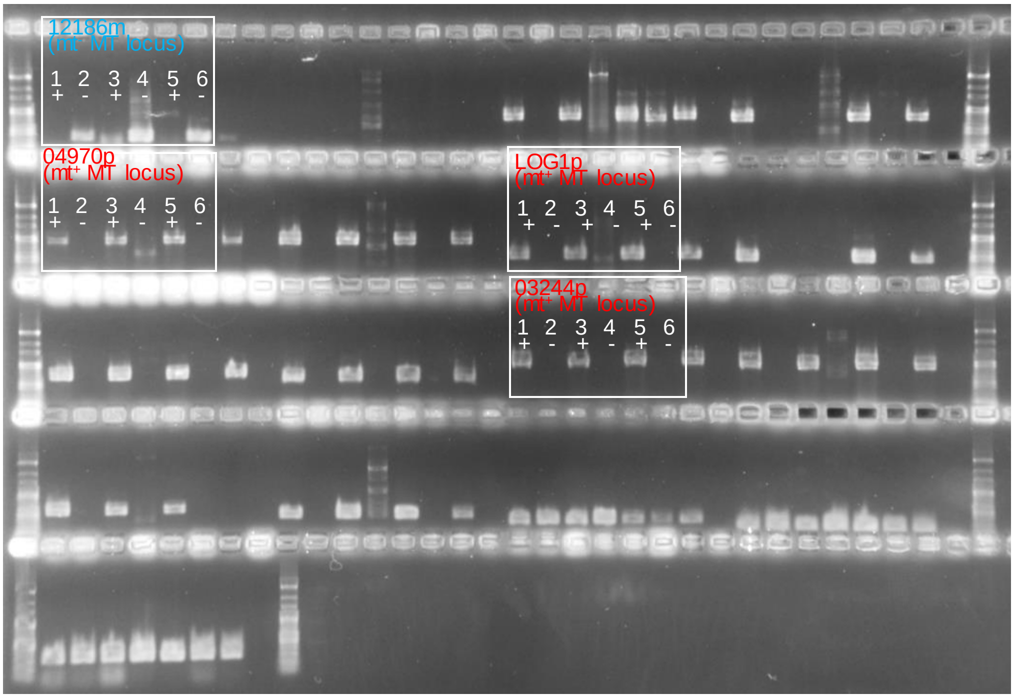

12186m
(mt- MT locus)
1 2 3 4 5 6
+ - + - + -
04970p
(mt+ MT locus)
1 2 3 4 5 6
+ - + - + -
LOG1p
(mt+ MT locus)
1 2 3 4 5 6
+ - + - + -
03244p
(mt+ MT locus)
1 2 3 4 5 6
+ - + - + -

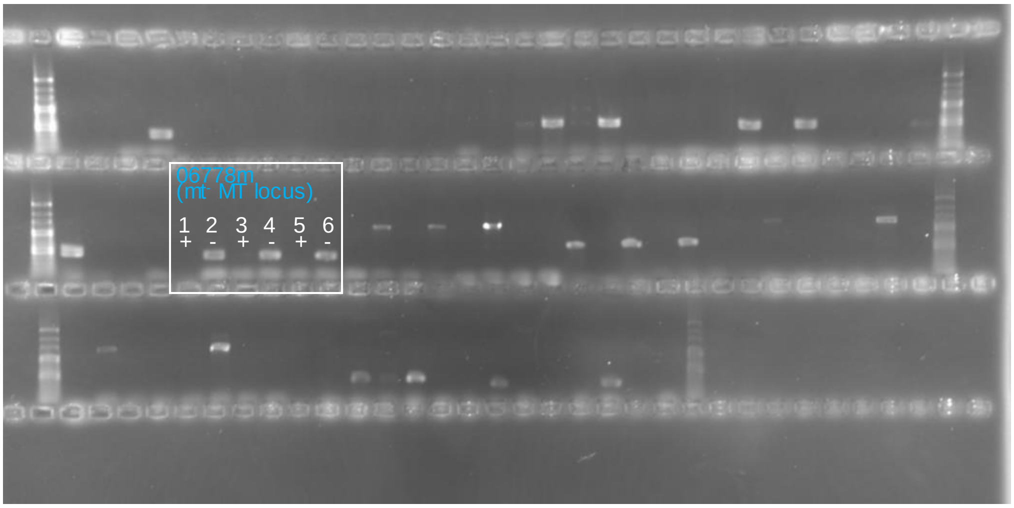

06778m
(mt- MT locus)
1 2 3 4 5 6
+ - + - + -

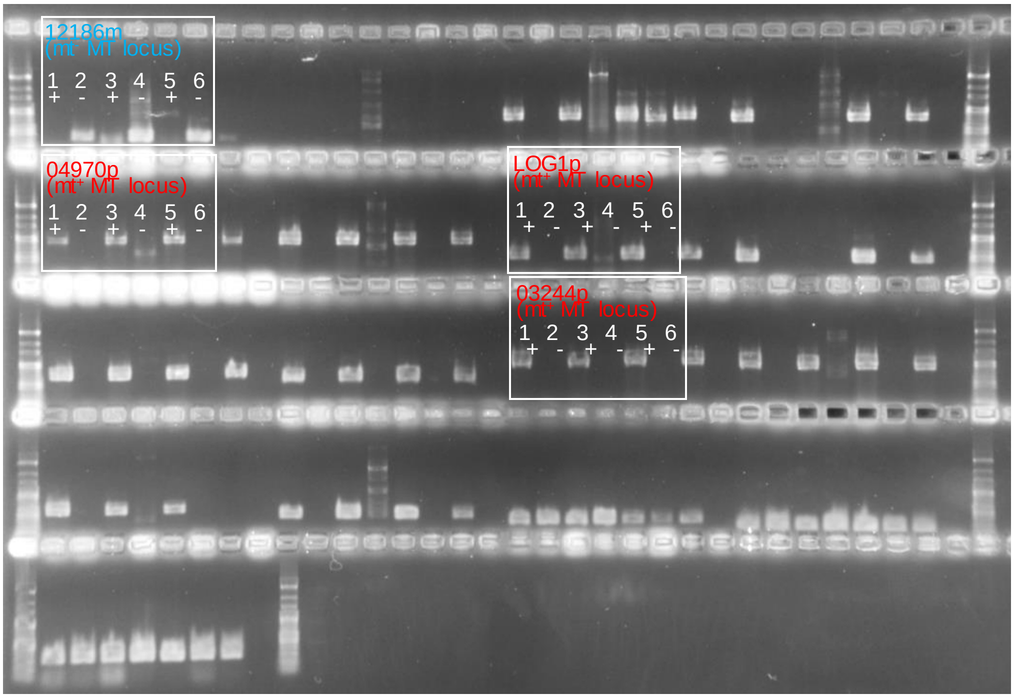

12186m
(mt- MT locus)
1 2 3 4 5 6
+ - + - + -
04970p
(mt+ MT locus)
1 2 3 4 5 6
+ - + - + -
LOG1p
(mt+ MT locus)
1 2 3 4 5 6
+ - + - + -
03244p
(mt+ MT locus)
1 2 3 4 5 6
+ - + - + -

Supplementary Figure 10. **Row data of fluorescence images of electrophoresed gels**.

Row data of fluorescence images of electrophoresed gels used in Figure 1B. The areas cropped for individual marker genes were shown by white boxes.

Supplementary Table 1. Summary of sequence data

Nucleotide	Sequence system	Strain	Mating type	Stage[4]	Read method	Total Read number[1] (x 10^6)	Sequence length[2] (bp)	Total sequence length[3] (Gbp)
DNA	PacBio RS	MGEC-2	mt^-	Gamete	-	1.7	7,182	12.1
		MGEC-1	mt^+	Gamete	-	2.7	6,136	16.6
	Illumina MiSeq	MGEC-2	mt-	Gamete	PE	271.1	100	27.1
		MGEC-1	mt^+	Gamete	PE	252.4	100	25.2
mRNA	Illumina HiSeq2500	MGEC-2	mt^-	Gametophyte	PE	60.8	100	5.9
		MGEC-2	mt^-	Gametophyte	PE	61.7	100	6.1
		MGEC-2	mt^-	Gametophyte	PE	38.6	100	3.7
		MGEC-2	mt^-	Gamete genesis (24h)	PE	36.8	100	3.6
		MGEC-2	mt^-	Gamete genesis (24h)	PE	30.4	100	3.0
		MGEC-2	mt^-	Gamete genesis (24h)	PE	32.1	100	3.1
		MGEC-2	mt^-	Gamete genesis (48h)	PE	47.5	100	4.6
		MGEC-2	mt^-	Gamete genesis (48h)	PE	38.3	100	3.7
		MGEC-2	mt^-	Gametegenesis (48h)	PE	31.9	100	3.1
		MGEC-2	mt^-	Gamete	PE	63.9	100	6.2
		MGEC-2	mt^-	Gamete	PE	66.5	100	6.5
		MGEC-2	mt^-	Gamete	PE	39.9	100	3.9
		MGEC-1	mt^+	Gametophyte	PE	59.2	100	5.8
		MGEC-1	mt^+	Gametophyte	PE	58.2	100	5.7
		MGEC-1	mt^+	Gametophyte	PE	42.2	100	4.1
		MGEC-1	mt^+	Gametophyte	PE	49.3	100	4.8
		MGEC-1	mt^+	Gamete genesis (24h)	PE	63.3	100	6.1
		MGEC-1	mt^+	Gamete genesis (24h)	PE	50.3	100	4.9
		MGEC-1	mt^+	Gamete genesis (24h)	PE	35.4	100	3.5
		MGEC-1	mt^+	Gamete genesis (48h)	PE	41.7	100	4.0
		MGEC-1	mt^+	Gamete genesis (48h)	PE	34.0	100	3.3
		MGEC-1	mt^+	Gametegenesis (48h)	PE	29.6	100	2.9
		MGEC-1	mt^+	Gamete	PE	61.8	101	6.0
		MGEC-1	mt^+	Gamete	PE	49.0	100	4.7
		MGEC-1	mt^+	Gamete	PE	42.2	100	4.1

[1]For PacBio, the numbers of only sub-reads for assemble (≥500bp)
[2]For PacBio, average read length
[3]For Illumina, reads through pass filter
[4]Time after induction of gamete genesis

Supplementary Table 2. Summary of mating type-specific PacBio reads

PacBio read type	Strain	Mating type	Read number	Average sequence length (bp)	Total sequence length (Mbp)
Total read	MGEC-2	mt^-	1,679,995	7,182	12,067
	MGEC-1	mt^+	2,703,640	6,136	16,591
Mating type specific read*	MGEC-2	mt^-	241	5,044	1.22
	MGEC-1	mt^+	320	4,676	1.50

*Unmapped PacBio reads on the opposite mating type scaffolds (less than 80% identity)

Supplementary Table 5. Gametologs in the MT locus

Gametolog Serial No.	Mating type				BLASTX E-value		Protein Identity (%)
	mt^-		mt^+				
	Gene name	Accession No.	Gene name	Accession No.			
1	03265m	LC088501	03265f	LC088620	2E-54	3E-54	25
2	PRP1m	LC088502	PRP1f	LC088626	6E-121	3E-121	83.87
3	HAR1m	LC088504	HAR1f	LC088671	3E-84	5E-85	98.46
4	ACTB1m	LC088548	ACTB1f	LC088670	9E-115	1E-114	95.33
5	CKK-CNB1m	LC088549	CKK-CNB1f	LC088639	0	0	84.35
6	ALP1m	LC088508	ALP1f	LC088590	1E-96	2E-96	81.5
7	12223m	LC088515	12223f	LC088600	7E-17	9E-17	50.82
8	MAPKKK1m	LC088555	MAPKKK1f	LC088664	0	0	88.61
9	LOG1m	LC088517	LOG1f	LC088634	9E-136	4E-136	84.62
10	SNR1m	LC088560	SNR1f	LC088608	2E-38	1E-31	85.14
11	SLG1m	LC088561	SLG1f	LC088606	3E-73	4E-73	64.33
12	PRA1m	LC088563	PRA1f	LC088633	1E-121	3E-122	80.13
13	06774m	LC088520	06774f	LC088661	3E-22	3E-17	85.94
14	03244m	LC088521	03244f	LC088623	2E-114	4E-115	82.09
15	05354m	LC088567	01940f	LC088677	2E-146	2E-146	93.26
16	DGK1m	LC088523	DGK1f	LC088595	2E-91	6E-106	70.43
17	10865m	LC088529	10865f	LC088613	6E-24	3E-23	-
18	PIK1m	LC088571	PIK1f	LC088632	9E-18	1E-18	45.95
19	12186m	LC088577	12186f	LC088593	8E-09	9E-09	-
20	SOM1m	LC088530	SOM1f	LC088652	3E-158	5E-144	66.79
21	eIF1m	LC088532	eIF1f	LC088588	0	0	88.89
22	02393m	LC088578	02393f	LC088597	0	0	88.52
23	MET1m	LC088534	MET1f	LC088647	0	0	94.55

Supplementary Table 6. Segregation of the *U. partita* MT locus genes among progeny of zoospores generated by the mating of two isolates

	Tester		Parent	Progeny															
Strain Name	EC2	EC5	EC2 x EC5	38	41	43	45	46	48	63	68	71	80	12	42	47	51	57	60
Result of matig type test	-	+	n.d.	-	-	-	-	-	-	-	-	-	-	+	+	+	+	+	+
07489m mt^{-}	+	-	+	+	+	+	+	+	+	+	+	+	+	-	-	-	-	-	-
06778m mt^{-}	+	-	+	+	+	+	+	+	+	+	+	+	+	-	-	-	-	-	-
02227p mt^{+}	-	+	+	-	-	-	-	-	-	+	-	-	-	+	+	+	+	+	+
03910p-1 mt^{+}	-	+	+	-	-	-	+	-	-	+	-	-	-	+	+	+	+	+	+
03910p-2 mt^{+}	-	+	+	-	-	-	+	-	-	+	-	-	-	+	+	+	+	+	+

Supplementary Table 7. List of specimens used in this study

Species	Sample ID	Mating type	Collection date	Location	Accession No. PRA1	Accession No. GTBP1	Reference
U. partita	MGEC-1	+	April 22, 1996	Futami-cho, Iyo city, Ehime perf., Japan.	LC088563	-	Kagami et al., 2008a
	MGEC-2	-	April 22, 1996	Futami-cho, Iyo city, Ehime perf., Japan.	LC088633	LC088700	Kagami et al., 2008a
	MGEC-3	+	-	Shishigawago, Tokitsu cho, Nagasaki perf., Japa	-	-	Kagami et al., 2008b
	MGEC-4	-	-	Shishigawago, Tokitsu cho, Nagasaki perf., Japa	-	-	Kagami et al., 2008b
	MGEC-5	+	October 30, 2004	Ofunawatari city, Iwate perf., Japan.	-	-	Kagami et al., 2008a
	MGEC-6	-	October 30, 2004	Ofunawatari city, Iwate perf. Japan.	-	-	Kagami et al., 2008a
U. compressa	UC12 F	+	September 23, 2012	Tralee bay holiday park, Oban, United Kingdom	LC088685	LC088693	Provided by Dr. Hiraoka, Koshi University
	UC13 M	-	September 23, 2012	Tralee bay holiday park, Oban, United Kingdom	-	LC088694	Provided by Dr. Hiraoka, Koshi University
U. fasciata	#1 F	+	May 11, 1999	Usa, Kochi pref., Japan.	LC088683	-	Hiraoka et al. 2003
	#1 M	-	May 11, 1999	Usa, Kochi pref., Japan,	LC088684	-	Hiraoka et al. 2003
U. meridionalis	E16 F	+	July 18, 2000	Tokushima, Tokushima pref., Japan.	LC088689	LC088697	Shimada et al. 2008
	E16 M	-	July 18, 2000	Tokushima, Tokushima pref., Japan.	LC088690	LC088698	Shimada et al. 2008
U. pertusa	#12 F	+	May 1, 1997	Usa, Kochi pref. Japan,	LC088682	LC088691	Shimada et al. 2003
	#11 M	-	May 1, 1997	Usa, Kochi pref. Japan,	-	LC088692	Shimada et al. 2003
U. prolifera	E21 F	+	February 25, 2001	Shimanto, Kochi pref., Japan	LC088688	LC088699	Shimada et al. 2008
	E21 M	-	February 25, 2001	Shimanto, Kochi pref., Japan	LC088686	-	Shimada et al. 2008
Ulva sp.	US3-3_F	+	May 12, 2014	Xiangshan, Zhejiang, China	LC088687	LC088696	Provided by Dr. Hiraoka, Koshi University
	US3-3_M	-	May 12, 2014	Xiangshan, Zhejiang, China	LC088681	LC088695	Provided by Dr. Hiraoka, Koshi University

Supplementary Table 8. Genes in the MT locus of *U. partita* and other green algae

Species	Strain	Mating type	Accession number	MT Genes/ CDS	Gametologs	Reference
Ulva partita	MGEC-2	mt^-		46	23	This study
Ulva partita	MGEC-1	mt^+		67	23	This study
Chlamydomonas reinhardtii	CC-2290	mt^-	GU814015	41	36	Science 328: 351–354; PloS Genet. 2013 9(8):e1003724.
Chlamydomonas reinhardtii	CC-503	mt^+	GU814014	40	36	Science 328: 351–354; PloS Genet. 2013 9(8):e1003724.
Volvox carteri	Adm, UTEX 1886	male	GU784916	70	46	Science 328: 351–354.
Volvox carteri	Eve, UTEX 1885	female	GU784915	80	46	Science 328: 351–354.

Supplementary Table 13. Averages of dN, dS, and dN/dS values for three species

Species	Method	dN	dS	dN/dS
Chlamydomonas reinhardtii	MS	0.01 ± 0.01	0.02 ± 0.04	0.18 ± 0.17
	MYN	0.01 ± 0.01	0.02 ± 0.04	0.16 ± 0.21
Volvox carteri	MS	0.12 ± 0.11	1.06 ± 1.05	0.22 ± 0.21
	MYN	0.12 ± 0.11	0.99 ± 0.98	0.24 ± 0.23
Ulva partita	MS	0.21 ± 0.18	1.30 ± 2.87	0.36 ± 0.16
	MYN	0.28 ± 0.21	1.39 ± 3.06	0.40 ± 0.16

Supplementary Table 16. Primer sets for MT locus genes

Gene name	Primer name	Sequence	Product size (bp)	Gene position			
07489m	Minus-07489-F1	TCACGTGATCAACTTGGTGA	362	Sf.632	1,264,420	1,264,905	+
	Minus-07489-R1	GCGTCTGGCTTGACCATAAT					
06377m	Minus-06377-F1	CTGGGATGATACAGTGAGCA	709	Sf.632	1,259,471	1,260,193	-
	Minus-06377-R1	CAGGGTGATGTGAGTTGTGA					
06778m	Minus-06778-F1	ACAGACAGTCATACATTGGGACC	399	Sf.632	2,041,813	2,042,802	-
	Minus-06778-R1	CAGGTAATGCCAACAGCCACTTG					
12186m	Minus-07576-F1	TAGAGTCTGTTGTTGAAGGCAGC	408	Sf.632	1,822,532	1,823,454	+
	Minus-07576-R1	AAAGCTTGGACTTCAAGATGCGG					
02227f	Plus-02227-F1	GATTCCCAGATTACCCGAAC	326	Sf.898	591,114	592,771	-
	Plus-02227-R1	CTTGCCGACTAATCCcTTGA					
04970f	Plus-04970-F2	GCAATGTTGTTTCCTTGGCCTGA	917	Sf.898	406,626	407,629	+
	Plus-04970-R2	GTGACCATGACCTGACATGCAAC					
LOG1f	Plus-00364-F1	TTATCTTGTACCCGCAAGTTGCC	555	Sf.4	452,036	454,975	-
	Plus-00364-R1	AGATTTACGGGATCACTGAAGCC					
03244f	Plus-01413-F2	ATTGTGTGTTCACAACAGGCAGG	916	Sf.4	82,240	84,905	-
	Plus-01413-R2	CTTTTCGCTGTTGCTCTAGCAGG					
